# Nanoimprint lithography for scalable electronic and photonic nanomanufacturing

**DOI:** 10.1186/s11671-026-04870-6

**Published:** 2026-09-21

**Authors:** Pei-Tien Chen, Chien-Chi Huang, Yu-Wen Lai, Chung-Hsiang Lin, Kang-Yuan Lee, Chia-Chen Li, Chih-Ting Chang, Chia-Jung Tsai, Li-Yin Chen, Po-Tsung Lee, Yu-Heng Hong, Tzu-Yi Lee, Hao-Chung Kuo

**Affiliations:** 1 Department of Photonics, Institute of Electro-Optical Engineering, College of Electrical and Computer Engineering, National Yang Ming Chiao Tung University, Hsinchu, 300093 Taiwan; 2 Semiconductor Research Center, Hon Hai Research Institute, Taipei, 114699 Taiwan; 3 Quantum NIL Corporation, Hsinchu, 300046 Taiwan; 4 Department of Electronic Engineering, Chung Yuan Christian University, Taoyuan, 320314 Taiwan

## Abstract

Nanoimprint lithography (NIL) has emerged as a powerful nanofabrication technology capable of bridging the gap between nanoscale precision and scalable manufacturing. Unlike conventional projection lithography, whose resolution is fundamentally constrained by diffraction and increasingly complex optical systems, NIL relies on direct mechanical replication of nanoscale patterns from a structured mold, enabling deterministic pattern transfer with sub-10 nm resolution. Over the past three decades, NIL has evolved from a laboratory-scale patterning concept into a technologically mature platform with growing industrial relevance across photonics, electronics, and large-area functional materials. This review provides a comprehensive overview of nanoimprint lithography, covering its historical development, technological evolution, material considerations, and emerging applications. By connecting NIL process mechanisms and material constraints with application-specific manufacturing requirements, this review provides a process-to-application perspective on scalable electronic and photonic nanomanufacturing. The fundamental principles and major NIL variants, including thermal NIL (T-NIL), ultraviolet NIL (UV-NIL), step-and-flash (S-FIL)/jet-and-flash (J-FIL) imprint lithography, and roll-to-roll NIL (R2R NIL), are systematically discussed with emphasis on process mechanisms, scalability, and manufacturing compatibility. Critical material considerations such as mold design, imprint resist properties, and substrate compatibility are also analyzed to elucidate the key parameters governing pattern fidelity, residual layer control, and process reliability. Particular attention is devoted to emerging application domains where NIL offers unique advantages, including nanophotonic metasurfaces, integrated silicon photonics, optoelectronic devices, and large-area optical structures. Recent demonstrations of high-efficiency metalenses, low-loss silicon photonic waveguides, and scalable flexible photonic components highlight the ability of NIL to combine subwavelength structural control with wafer-scale replication. As demands for advanced photonic integration and functional nanostructures continue to increase, nanoimprint lithography is increasingly recognized as a scalable manufacturing pathway capable of translating nanoscale device concepts into practical technologies.

## Introduction and historical evolution of NIL

### Origin and motivation of nanoimprint lithography

Nanoimprint lithography (NIL) was proposed in the mid-1990s as an alternative nanofabrication approach in response to the increasing physical and economic limitations of conventional optical lithography. As device dimensions continued to shrink toward the deep sub-wavelength regime, projection-based lithography encountered fundamental challenges. Diffraction limits restricted achievable resolution, while optical proximity effects increasingly complicated pattern fidelity at the nanoscale. Meanwhile, advanced lithography technologies required extremely sophisticated optical systems, multi-patterning processes, and rapidly escalating capital investment. These limitations motivated the search for alternative patterning strategies capable of achieving nanoscale resolution while reducing process complexity and manufacturing cost. NIL provides a fundamentally different route to nanoscale patterning. Instead of defining structures through optical projection or electron-beam writing, NIL transfers nanoscale patterns through direct mechanical replication between a patterned mold and a deformable resist layer. This concept was first demonstrated by Chou and co-workers in 1995, who showed that sub-25 nm features could be replicated in thermoplastic polymers without dependence on exposure wavelength [1]. The sub-25 nm value refers specifically to the original 1995 demonstration, whereas subsequent advances in template fabrication, resist materials, and imprint process control have enabled NIL to achieve sub-10 nm resolution. By eliminating the wavelength constraint inherent to optical lithography, NIL bypasses diffraction limits and enables pattern definition primarily determined by template fidelity and resist flow behavior [2]. This represented a conceptual shift from exposure-defined lithography to template-defined replication. Once a high-quality template is fabricated, nanoscale structures can be repeatedly replicated with high structural fidelity through a relatively simple process architecture, enabling deterministic nanostructure fabrication. Beyond its high-resolution capability, NIL also offers significant advantages in manufacturing scalability. Because imprinting is intrinsically a parallel replication process, entire pattern fields can be transferred in a single step, in contrast to serial writing techniques such as electron-beam lithography [3]. This parallel nature enables high-throughput nanomanufacturing while maintaining nanoscale precision. Over the past two decades, several NIL variants have been developed to improve manufacturability and expand application opportunities across diverse nanofabrication fields. These approaches address key practical challenges such as residual layer thickness (RLT) control, defect mitigation, overlay alignment, and large-area pattern transfer, enabling NIL to gradually transition from laboratory demonstrations toward industrial manufacturing technologies. The technology is capable of producing nanostructures with dimensions down to the nanometer scale while maintaining relatively low cost and high throughput, making it attractive for large-area optical and electronic devices [4]. In recent years, growing demand for advanced nanofabrication technologies in semiconductor, photonic, and optoelectronic applications has renewed industrial interest in NIL. Improvements in imprint tools, template fabrication, and resist materials have further expanded its potential for scalable manufacturing of functional nanostructures [5]. Several review articles have previously discussed NIL process technologies, materials, and specific application domains. Rather than focusing on a single aspect of NIL, this review provides an integrated perspective on the evolution of NIL, major process variants, manufacturing considerations, and emerging applications in both photonic and semiconductor devices. Within this scope, photonic and optoelectronic implementations are treated broadly, while electronic nanomanufacturing is addressed through representative NIL-enabled semiconductor structures and emerging CMOS-relevant device applications. Particular emphasis is placed on how different NIL approaches address the requirements of scalable nanomanufacturing and bridge the gap between laboratory-scale nanopatterning and industrial implementation.

Consequently, the motivation for NIL today extends far beyond overcoming diffraction limits. Its importance lies in bridging nanoscale structural precision with scalable manufacturing infrastructure. As emerging technologies continue to demand deterministic nanostructures with high reproducibility across large areas, nanoimprint lithography offers a promising approach to combining nanoscale precision, manufacturing scalability, and economic viability [6]. This combination positions NIL as a key enabling platform for next-generation nanophotonic, optoelectronic, and semiconductor device fabrication.

### Key milestones from laboratory demonstrations to industrial adoption

**Fig. 1:**
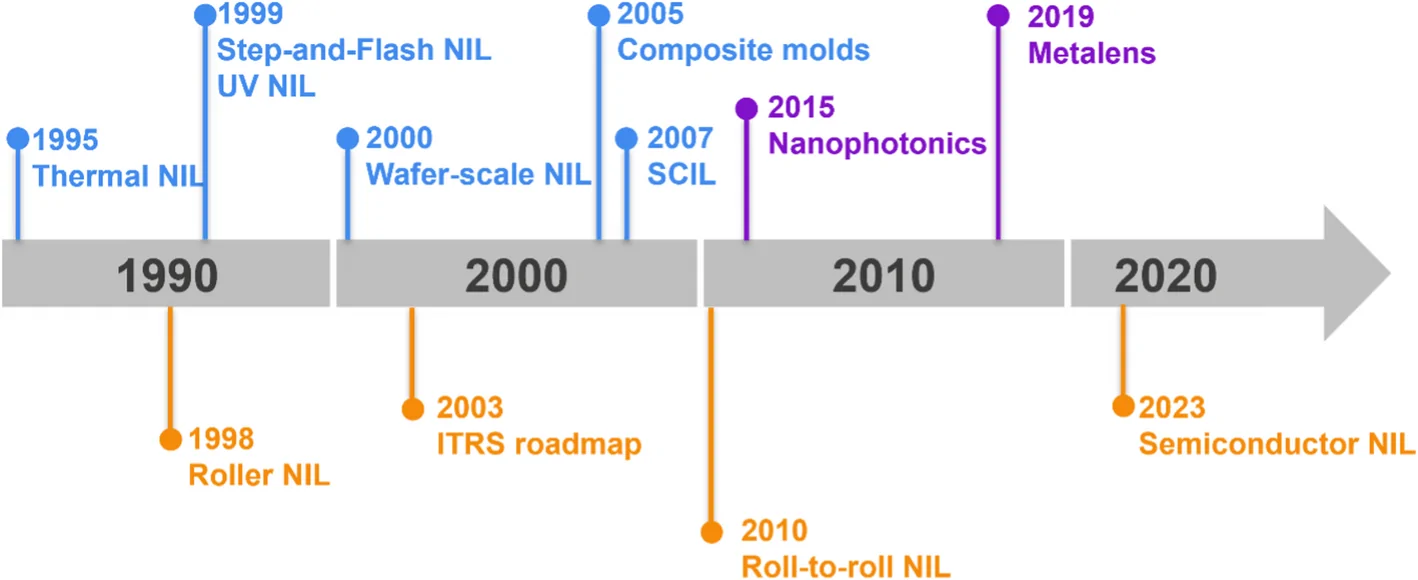
Development timeline of nanoimprint lithography, highlighting major milestones from early thermal imprinting and wafer-scale processing to scalable photonic and semiconductor manufacturing. Created by the authors based on the literature cited in Sect.  1.2

Since its first demonstration in 1995, nanoimprint lithography (NIL) has gradually evolved from a laboratory-scale pattern replication method into a versatile nanofabrication approach with growing relevance for practical manufacturing. As illustrated in Fig. 1, the development of NIL over the past three decades reflects a progression from early proof-of-concept experiments toward wafer-scale processing and, more recently, toward the fabrication of increasingly complex functional nanostructures. Early work demonstrated that nanoscale patterns could be mechanically transferred into thermoplastic polymers using patterned molds, establishing the fundamental concept of imprint-based lithography [7]. Subsequent developments such as S-FIL and ultraviolet NIL further expanded the versatility of the technique, enabling pattern transfer through UV-curable resists and improving processing flexibility. During this stage, research mainly focused on understanding the physical mechanisms governing the imprint process, including polymer flow behavior, mold fabrication precision, and residual layer control, thereby establishing the basic principles of imprint-based nanofabrication [2, 8].

As the technology matured in the early 2000s, NIL began moving beyond small-scale demonstrations toward more practical fabrication strategies. Wafer-scale imprinting experiments demonstrated that nanoscale patterns could be replicated over several-inch substrates, marking an important step toward scalable nanomanufacturing. Around the same time, early concepts such as roller-based nanoimprint lithography illustrated the possibility of continuous pattern replication, while the inclusion of NIL in the International Technology Roadmap for Semiconductors (ITRS) further highlighted its potential as a cost-effective alternative for certain high-resolution patterning applications. These developments indicated that imprint-based techniques could extend beyond laboratory studies and potentially serve as viable nanofabrication technologies for larger-scale production [7, 9]. Further improvements during the mid-2000s focused on enhancing process reliability and pattern uniformity. Composite mold technologies and substrate conformal imprint lithography (SCIL) were introduced to address practical challenges such as mold durability, demolding stability, and defect formation during large-area replication. By improving mold compliance and contact uniformity, these approaches significantly enhanced the reproducibility of NIL processes and enabled more reliable pattern transfer across larger substrates. Entering the 2010s, NIL technologies began to intersect more strongly with emerging nanophotonic applications. Increasing interest in metasurfaces, photonic nanostructures, and other functional optical devices created a growing demand for fabrication techniques capable of producing sub-wavelength structures over large areas [10]. During this period, imprint-based methods were increasingly explored as scalable alternatives to conventional electron-beam lithography for the fabrication of nanophotonic structures. Continuous processing concepts such as roll-to-roll nanoimprint lithography further demonstrated the potential for high-throughput nanopatterning on flexible substrates, highlighting the compatibility of NIL with large-area manufacturing approaches [11, 12].

More recently, NIL has continued moving toward practical industrial deployment. Improvements in imprint equipment, template fabrication, and resist materials have enhanced process stability and overlay accuracy, allowing NIL to be integrated more readily with conventional semiconductor manufacturing workflows. Recent demonstrations of semiconductor-oriented NIL systems operating on 12-inch wafers indicate that the technology has progressed well beyond early laboratory experiments [13, 14]. At the same time, the successful fabrication of advanced nanophotonic devices such as metalenses illustrates how NIL can support the scalable production of complex optical nanostructures. Together, these developments show how nanoimprint lithography has gradually transitioned from an early high-resolution patterning concept into a practical nanomanufacturing platform capable of replicating functional nanostructures across large areas.

### Distinct roles of NIL in photonics versus conventional semiconductor manufacturing

Although NIL was initially proposed as a potential alternative to conventional semiconductor lithography, its practical role in modern nanofabrication has gradually diverged from that of projection-based technologies used in CMOS manufacturing. In conventional semiconductor fabrication, lithography is primarily used to define transistor gate patterns and critical device layers that require extremely precise overlay alignment across multiple process steps. Technologies such as deep ultraviolet (DUV) and extreme ultraviolet (EUV) lithography therefore focus on achieving nanometer-scale overlay accuracy, high pattern fidelity, and tight integration with complex multilayer CMOS process flows. In contrast, NIL operates through direct mechanical replication between a patterned template and a deformable resist layer, which fundamentally changes how pattern definition is achieved. Because pattern transfer is achieved through direct template replication rather than optical projection, NIL exhibits distinct manufacturing characteristics compared with conventional projection lithography [8].

This difference in patterning mechanism leads to distinct application domains for NIL and conventional semiconductor lithography. In the semiconductor industry, NIL faces challenges when applied to advanced CMOS logic processes, particularly due to requirements for sub-nanometer overlay alignment and extremely low defect densities. For advanced CMOS manufacturing, overlay tolerances of only a few nanometers and extremely low defect densities remain critical requirements that continue to challenge NIL implementation. In contrast, the performance of many photonic devices is primarily governed by nanoscale structural geometry rather than multilayer alignment accuracy, making NIL particularly attractive for metasurfaces, metalenses, photonic crystals, and diffractive optical components [10]. As a result, NIL has found particularly strong adoption in nanophotonic devices where deterministic nanoscale structures must be replicated across large areas. Typical examples include metasurfaces, metalenses, photonic crystals, diffractive optical elements, and light-management layers for photovoltaic and display technologies. In these applications, the ability of NIL to replicate complex nanostructures with high fidelity and over large substrate areas becomes a significant advantage compared with serial lithographic techniques [15]. The requirements of these devices differ fundamentally from those of advanced CMOS logic, where overlay accuracy and defect control dominate process integration. In many photonic and optoelectronic systems, device performance is governed primarily by nanoscale structural geometry, making large-area pattern replication a more critical consideration than multilayer alignment precision. This difference has contributed to the growing adoption of NIL in metasurface optics, photonic devices, and other emerging nanostructured platforms.

Another important distinction lies in manufacturing scalability and process economics. Conventional projection lithography relies on extremely sophisticated optical systems and high-energy radiation sources, which significantly increase tool cost and energy consumption. NIL, by contrast, operates through a relatively simple mechanical imprinting process that allows entire pattern fields to be transferred in a single step. This inherently parallel nature enables high-throughput replication once a master template has been fabricated. As summarized in Table 1, NIL provides a different balance of characteristics compared with conventional lithography technologies, particularly in terms of pattern formation mechanism, manufacturing scalability, and process economics. While projection-based lithography remains essential for multilayer transistor fabrication requiring high overlay precision, NIL provides unique advantages for applications that require nanoscale structural control across large areas but involve fewer alignment-critical process steps.

**Table 1:** Comparison of photolithography (PhL), electron-beam lithography (EBL), and NIL in terms of pattern formation mechanism, resolution capability, process characteristics, manufacturing efficiency, and cost considerations

Comparison parameter	Photolithography (PhL)	Electron-Beam Lithography (EBL)	Nanoimprint Lithography (NIL)
Pattern formation mechanism	Patterns are defined through optical exposure and photoresist development	Nanoscale structures are directly written by a focused electron beam	Nanostructures are replicated through mechanical contact between a patterned mold and resist
Achievable resolution	Resolution is limited by exposure wavelength and optical system design, typically reaching tens of nanometers in advanced systems	Direct writing enables feature sizes down to a few nanometers	Pattern dimensions are primarily determined by mold fidelity and can reach sub-10 nm replication
Process characteristics	Parallel exposure process suitable for wafer-scale semiconductor fabrication	Serial writing process, which limits efficiency for large-area patterning	Parallel replication process capable of transferring patterns over large areas in a single imprint step
Manufacturing efficiency	Pattern transfer can be completed rapidly during exposure and development steps	Pattern generation is relatively slow due to point-by-point beam scanning	Imprinting and curing steps are typically completed within seconds to minutes
Cost considerations	Advanced optical lithography systems require complex equipment and high capital investment	High equipment and operational costs, mainly used for research or mask fabrication	Equipment architecture is relatively simple, offering potential cost advantages for large-area nanofabrication
References	[2, 9, 16]	[2, 3]	[3, 8]

Consequently, NIL should not be viewed simply as a direct replacement for conventional semiconductor lithography. Instead, it plays a complementary role in the broader nanofabrication ecosystem. Projection-based lithography continues to dominate advanced logic and memory manufacturing, whereas NIL is increasingly positioned as a scalable fabrication technology for nanophotonic and optoelectronic devices [17]. This complementary relationship highlights how NIL can bridge the gap between nanoscale structural precision and large-area manufacturability, enabling device architectures that would otherwise be difficult or prohibitively expensive to fabricate using conventional semiconductor lithography alone.

## Principles of nanoimprint lithography and technology variants

### Fundamental concept of nanoimprint lithography

NIL is a nanoscale patterning technique fundamentally based on direct mechanical replication. Unlike projection-based lithographic approaches, where structural definition relies on photon or electron exposure, NIL forms patterns through physical contact between a patterned mold and a deformable resist layer. In other words, instead of defining structures through exposure chemistry, NIL directly replicates the geometry defined by the mold itself. This replication-based concept can be understood through three main stages: resist preparation, imprinting, and pattern transfer [2]. In a typical process, a thin resist layer, either thermoplastic or photo-curable, is first deposited onto the substrate. A mold containing nanoscale relief structures is then pressed into the resist under controlled pressure so that the resist conforms to the mold geometry. Depending on the process variant, this deformation occurs either through viscoelastic flow at elevated temperature or through photo-induced polymerization at room temperature [16]. After the resist solidifies or cools, the mold is separated, leaving a negative replica of the original pattern in the resist layer. The replicated structure can then be transferred into the underlying functional material through residual layer removal and anisotropic etching.

Because NIL relies on physical replication rather than optical exposure, its resolution is primarily determined by template fidelity and resist deformation behavior rather than by exposure wavelength or beam size. This fundamental difference distinguishes NIL from diffraction-limited projection lithography. In principle, if a master mold contains sub-10 nm features and the resist can fully fill the mold cavities without structural collapse during demolding, these dimensions can be faithfully reproduced in the imprinted resist. Achieving such fidelity, however, requires careful control of several process parameters. Mold fabrication precision, resist viscosity, capillary or pressure-driven filling dynamics, and interfacial adhesion during mold separation all play important roles in determining the final pattern quality [17]. Another inherent characteristic of imprint-based processing is the formation of a residual layer beneath patterned structures. Because the resist is initially coated as a continuous thin film, cavity filling typically leaves a thin interconnected base layer between adjacent features. The thickness and uniformity of this residual layer strongly influence etching selectivity, dimensional control, and defect formation during pattern transfer [18]. As a result, process optimization often requires coordinated control of resist thickness, imprint pressure, temperature or curing dose, and mold geometry to achieve uniform filling while minimizing residual layer variation.

From a process perspective, NIL techniques are generally categorized according to the dominant solidification mechanism, namely thermally induced deformation or photo-initiated curing. Although these two approaches differ in materials, processing temperature, and cycle time, both rely on the same replication-centered framework. Another important feature of NIL is that it operates as a parallel replication technique. Instead of writing patterns one feature at a time, as in electron-beam lithography, the entire pattern field can be transferred in a single imprint step. Consequently, the scalability and reproducibility of NIL depend less on optical projection constraints and more on factors such as mold durability, defect mitigation, and alignment precision [5]. This replication-driven concept has enabled the development of various NIL process variants over the past decades and provides the conceptual foundation for understanding the technological evolution discussed in the following sections.

### Core process architectures of nanoimprint lithography

#### Thermal nanoimprint lithography

T-NIL, also referred to as hot embossing lithography, is a thermomechanical pattern transfer technique in which nanoscale features are replicated by deforming thermoplastic polymers above their glass transition temperature (Tg). Unlike projection-based lithography, T-NIL does not rely on optical exposure; instead, structural information is transferred through direct mechanical contact between a rigid mold and a softened polymer layer. Because the process depends on physical deformation rather than diffraction-limited exposure, nanoscale structures can be replicated with very high fidelity. In a typical process, a thermoplastic polymer film is first heated above Tg, where the material transitions from a rigid glassy state into a viscoelastic state with significantly reduced viscosity. Under applied pressure, the softened polymer flows into the nanoscale cavities of the mold. Once the polymer fills the mold features, the system is cooled below Tg so that the material solidifies and preserves the replicated geometry before demolding [2, 17, 19].

**Fig. 2:**
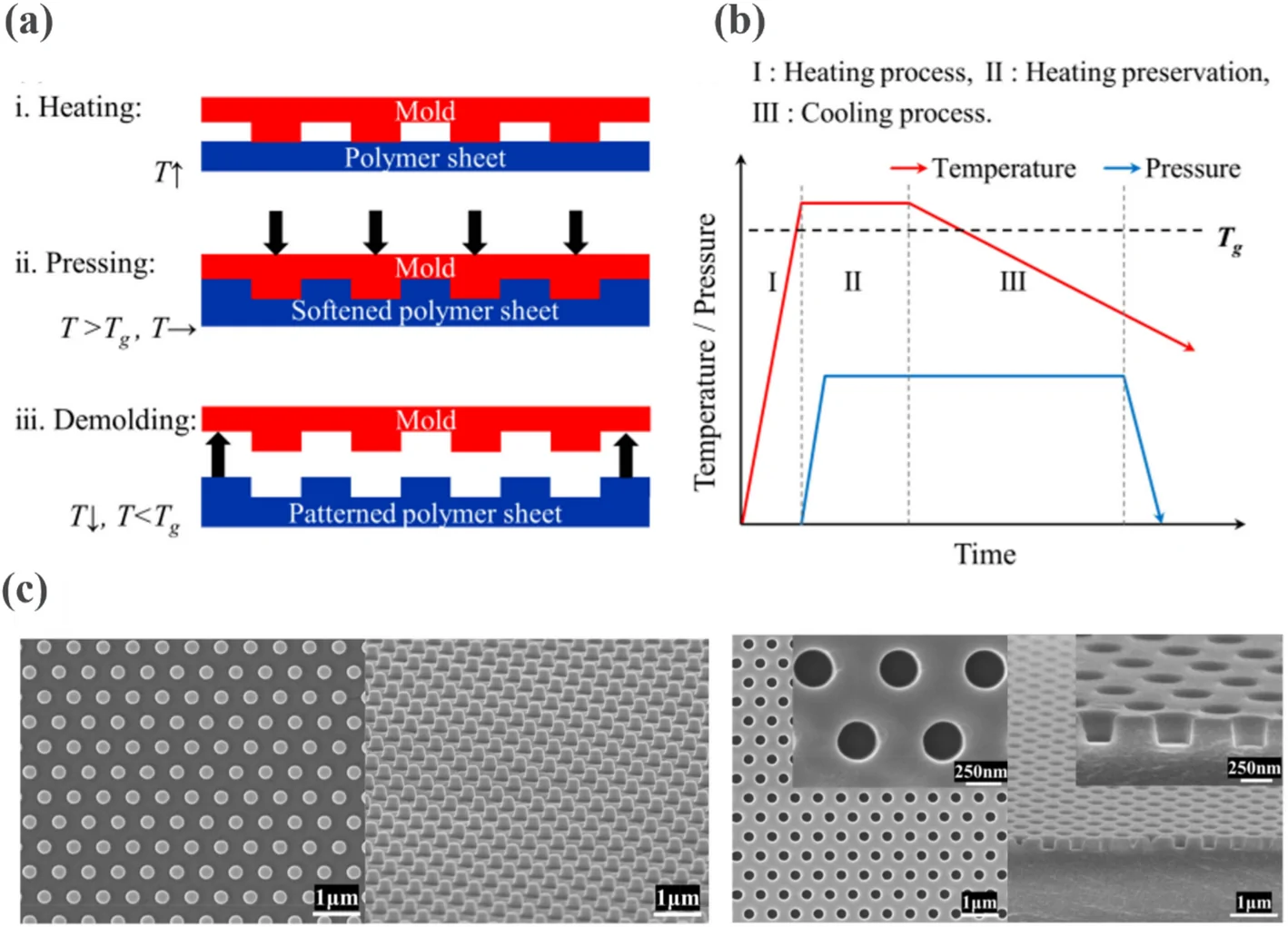
T-NIL process and structural fidelity. **a** Schematic of heating, pressurization, and cooling cycle. **b** Temperature–pressure evolution during imprinting. **c** SEM images comparing quartz master gratings and imprinted polymer replicas, demonstrating high-fidelity pattern transfer. Adapted from Ref [22]. with permission

As schematically illustrated in Fig. 2a, the T-NIL process can be described through three main stages: heating, pressurization, and cooling, followed by mold separation. During heating (T > Tg), polymer viscosity decreases by several orders of magnitude, enabling viscous flow. External pressure then drives the polymer into nanoscale recesses of the mold during the pressing stage. Cooling under sustained pressure subsequently suppresses structural relaxation and stabilizes the imprinted geometry before the mold is released. The thermal–mechanical cycle governing this process is summarized in Fig. 2b, which illustrates the evolution of temperature and pressure during imprinting. In practice, the imprinting sequence typically includes temperature ramp-up above Tg, pressure holding at elevated temperature to ensure complete cavity filling, and controlled cooling under load. The coupling between temperature, pressure, and time therefore plays a critical role in determining replication fidelity and RLT. Studies published after 2010 have examined polymer flow behavior during embossing and show that cavity filling is mainly governed by polymer viscosity, cavity geometry, and applied stress [20, 21]. Two primary filling modes are commonly observed: squeeze flow initiated from cavity edges and localized vertical filling in confined regions. These flow dynamics strongly influence filling time, residual layer uniformity, and structural homogeneity across large areas.

From a materials perspective, T-NIL also offers advantages when fabricating monolithic thermoplastic substrates because no additional photo-curable resist layer is required [17]. This reduces potential shrinkage or mechanical mismatch associated with UV-curable systems. Consequently, T-NIL has been widely applied in nanophotonic gratings, anti-reflection surfaces, flexible electronics, and light-management structures for thin-film photovoltaics [23]. Despite these advantages, reliable demolding remains a critical challenge, particularly for high-aspect-ratio structures that are susceptible to adhesion-induced deformation or fracture during separation. To address this issue, surface engineering strategies such as anti-adhesion coatings and bio-inspired polymer interlayers have been developed to reduce interfacial adhesion and demolding stress, thereby improving imprint uniformity and defect control in T-NIL systems [24]. Overall, by optimizing polymer rheology, thermal cycling conditions, and interfacial adhesion control, T-NIL provides a robust and scalable platform for high-resolution nanoscale pattern replication.

#### Ultraviolet nanoimprint lithography (UV-NIL)

UV-NIL is a room-temperature pattern replication technique that transfers nanoscale features using UV-curable resists under relatively low imprint pressure [18]. Unlike T-NIL, which relies on thermoplastic deformation above the glass transition temperature, UV-NIL solidifies liquid resists through photopolymerization after the mold cavities are filled. Because the process avoids high-temperature cycling, thermal stress, processing time, and substrate distortion can be significantly reduced. This makes UV-NIL particularly suitable for fabricating large-area optical and electronic devices. In a typical process, a low-viscosity liquid resist is first spin-coated or dispensed onto the substrate. When a transparent mold contacts the resist, capillary forces drive the liquid into nanoscale cavities of the mold. After the cavities are filled, ultraviolet irradiation initiates crosslinking of the resist, transforming the liquid into a mechanically stable polymer network. The mold is then separated, leaving replicated nanostructures on the substrate surface.

**Fig. 3:**
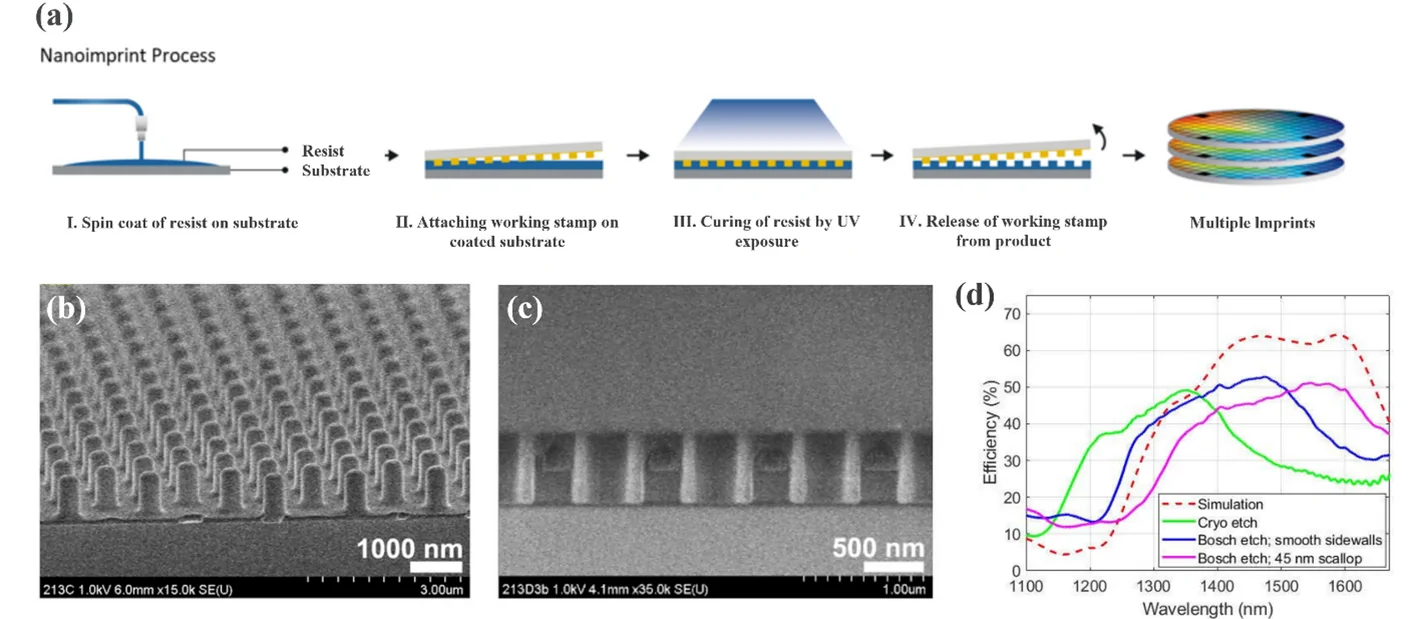
UV-NIL process and device-level validation. **a** Capillary-driven filling and UV curing sequence. Adapted from Ref [18]. with permission. **b** High-aspect-ratio nanopillar structures fabricated using UV-curable resist. Adapted from Ref [25]. with permission. **c** Cross-sectional SEM after CF₄ plasma etching showing pattern transfer into fused silica. Adapted from Ref [25]. with permission. **d** Measured and simulated optical efficiencies of metasurface structures fabricated using UV-NIL. Adapted from Ref [26]. with permission

The sequence of these steps is illustrated in Fig. 3a, which shows the typical UV-NIL process consisting of resist coating, capillary-driven filling under mold contact, and UV curing, followed by demolding. While T-NIL is governed by a thermomechanical cycle, the behavior of UV-NIL is mainly determined by the interplay between resist viscosity, surface energy, capillary pressure, and UV exposure dose. Filling behavior is primarily driven by capillary forces rather than externally applied mechanical stress. Studies have shown that successful cavity filling strongly depends on resist viscosity, cavity geometry, RLT, and mold–resist surface energy matching [25]. Air entrapment during mold contact may also generate bubble defects, particularly in large-area patterns or structures with complex cavity geometries, resulting in local pattern distortion and reduced replication fidelity. Incomplete filling may result in void formation or nonuniform residual layers, whereas excessive resist volume can increase demolding stress and defect probability. The structural fidelity achievable with UV-NIL is demonstrated in Fig. 3b, where scanning electron microscopy (SEM) images show imprinted nanopillar structures fabricated using optimized UV-resist formulations under ambient conditions. The replicated features exhibit heights of approximately 830–840 nm with well-defined sidewalls and minimal collapse, indicating that UV-NIL can replicate high-aspect-ratio nanostructures with high fidelity. Improved resistance to oxygen inhibition in advanced resist formulations also enables reliable curing when elastomeric molds are used under atmospheric conditions [25].

Etch durability and large-area process uniformity are also critical for practical device fabrication. Cross-sectional SEM images obtained after CF₄ plasma etching at 500 W, shown in Fig. 3c, demonstrate that the imprinted resist maintains structural integrity during fused silica etching. Reported etch selectivity values exceeding 0.6 under high-power inductively coupled plasma (ICP) conditions indicate that UV-cured resists can function as effective direct etch masks for glass substrates. The preservation of vertical sidewalls after plasma exposure reflects the mechanical stability and chemical resistance of the crosslinked polymer network. The device-level optical performance of structures fabricated through UV-NIL is illustrated in Fig. 3d. The measured optical efficiency spectra show good agreement with simulation results, confirming that the imprinted nanostructures maintain their designed optical functionality after pattern transfer and etching processes. These results demonstrate that UV-NIL can support the fabrication of functional nanophotonic devices while preserving the structural fidelity of the imprinted features [26]. The close agreement between simulation and experiment further suggests that uniform resist filling, controlled residual-layer thickness, and defect-minimized pattern transfer are essential for maintaining the designed optical response of the fabricated nanostructures.

Relative to T-NIL, UV-NIL typically provides shorter cycle times, lower energy consumption, and improved compatibility with temperature-sensitive substrates. Because the process avoids high-temperature cycling, thermal stress and substrate deformation can be significantly reduced while maintaining relatively short processing cycles. Dimensional shrinkage can also be controlled through appropriate resist formulation. Nevertheless, reliable demolding remains a key challenge, particularly for high-aspect-ratio nanostructures. Surface-energy engineering and anti-sticking layer optimization are commonly employed to reduce adhesion-induced deformation and defect formation [27]. Continued improvements in resist chemistry, capillary filling dynamics, curing kinetics, and interfacial adhesion control have allowed UV-NIL to become a versatile nanofabrication platform widely used in metasurfaces, nanophotonics, and glass-based device integration.

#### Jet-and-flash NIL

**Fig. 4:**
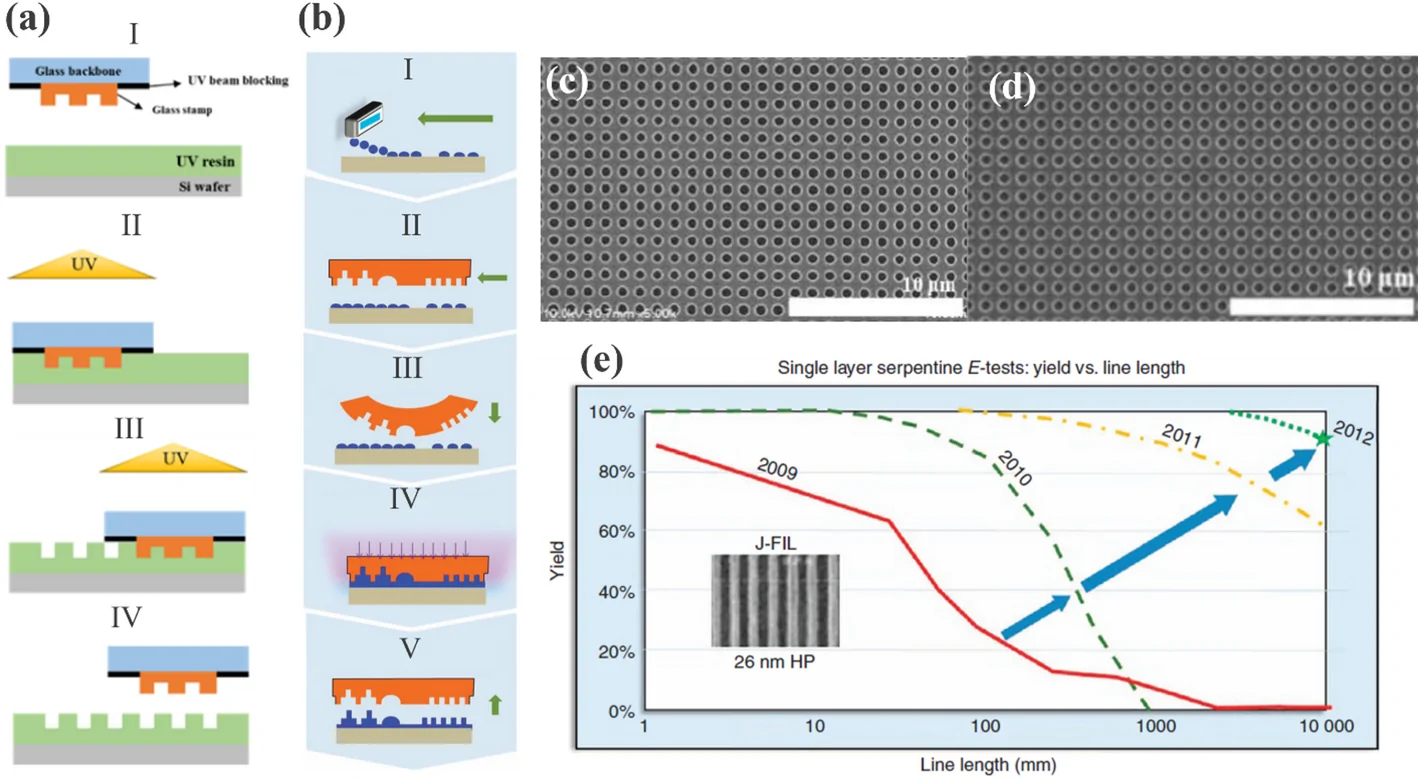
Stepper-based nanoimprint lithography for semiconductor integration. **a** Step-and-Flash imprint sequence. Adapted from Ref [29]. with permission. **b** Drop-on-demand resist dispensing in Jet-and-Flash imprint lithography. Adapted from Ref [5]. with permission. **c** SEM image of the nanopatterned template used in the step-and-repeat nanoimprint process. Adapted from Ref [29]. with permission. **d** Replicated nanostructures on the substrate demonstrating high-fidelity pattern transfer after nanoimprint lithography. Adapted from Ref [29]. with permission. **e** Electrical yield evolution of serpentine E-test structures demonstrating process maturity. Adapted from Ref [5]. with permission

S-FIL was originally introduced to adapt nanoimprint lithography to semiconductor-style wafer processing. In conventional S-FIL, a low-viscosity UV-curable resist is deposited onto the substrate before template contact, and pattern transfer is performed in a step-and-repeat manner using a transparent template and UV curing. Rather than imprinting an entire wafer in a single step, the template sequentially patterns localized regions across the wafer surface, similar to the operation of optical lithography steppers [28]. This configuration improves overlay control and reduces the risk of large-area defect propagation, both of which are critical for multilayer nanofabrication. The basic S-FIL sequence is illustrated in Fig. 4a. In practice, a small amount of resist is first dispensed onto the substrate, after which the template contacts the resist, and capillary forces drive the liquid into nanoscale cavities. Ultraviolet exposure then cures the resist, and the template is separated before the stage moves to the next imprint field. Because only a small template area is used at each step, the need for a full-wafer mold is avoided, reducing template fabrication complexity and cost. Careful control of stage positioning is also essential to suppress stitching errors between adjacent imprint fields. For instance, Ha et al. reported sub-micrometer stitching accuracy on 4-inch substrates using optimized stage displacement intervals and high-precision positioning systems [29]. As S-FIL evolved toward semiconductor manufacturing, resist-volume control and residual-layer thickness (RLT) uniformity became increasingly important. Variations in resist distribution can influence filling kinetics, generate nonuniform residual layers, and ultimately affect pattern transfer fidelity. To address these limitations, J-FIL was developed. Unlike conventional S-FIL, J-FIL employs drop-on-demand inkjet dispensing of UV-curable resist droplets immediately before template contact, as illustrated in Fig. 4b. The key distinction between S-FIL and J-FIL therefore lies in the resist delivery strategy rather than the imprint architecture, since both techniques utilize step-and-repeat UV imprinting. By precisely controlling resist volume at each imprint location, J-FIL promotes more uniform filling behavior, suppresses residual-layer nonuniformity, reduces material consumption, and minimizes bubble-related defects caused by air entrapment during mold contact. Industrial demonstrations summarized by Traub et al. have shown that J-FIL systems can achieve overlay accuracies approaching ~ 5 nm together with throughput levels compatible with semiconductor manufacturing requirements [10, 29]. These improvements have significantly enhanced process stability and yield, making J-FIL one of the most promising NIL approaches for semiconductor integration.

The replication capability of the step-and-repeat nanoimprint process is demonstrated in Fig. 4c and d. Figure 4c shows the SEM image of the nanopatterned template used in the step-and-repeat nanoimprint process, while Fig. 4d presents the corresponding replicated nanostructures on the substrate after imprinting. The imprinted features closely follow the geometry of the template, illustrating the one-to-one pattern transfer characteristic of nanoimprint lithography. After imprinting, the remaining residual resist layer is typically removed by anisotropic plasma etching to transfer the pattern into the underlying substrate [5]. The growing technological maturity of J-FIL is also reflected in electrical yield measurements. As shown in Fig. 4e, single-layer serpentine E-test structures exhibited substantial yield improvement between 2009 and 2012, eventually reaching nearly 100% yield for 26 nm half-pitch patterns over millimeter-scale line lengths.

These results reflect steady progress in defect control, contamination management, overlay precision, and template durability. With these developments, J-FIL has moved well beyond early laboratory demonstrations and is now considered a promising candidate for high-volume semiconductor manufacturing [5]. By combining stepper-level alignment, controlled droplet-based resist delivery, and high-fidelity template replication, S-FIL and J-FIL provide a practical pathway for bridging laboratory nanoimprint research with semiconductor-grade nanofabrication platforms [5, 30, 31].

#### Roll-to-roll nanoimprint lithography (R2R NIL)

**Fig. 5:**
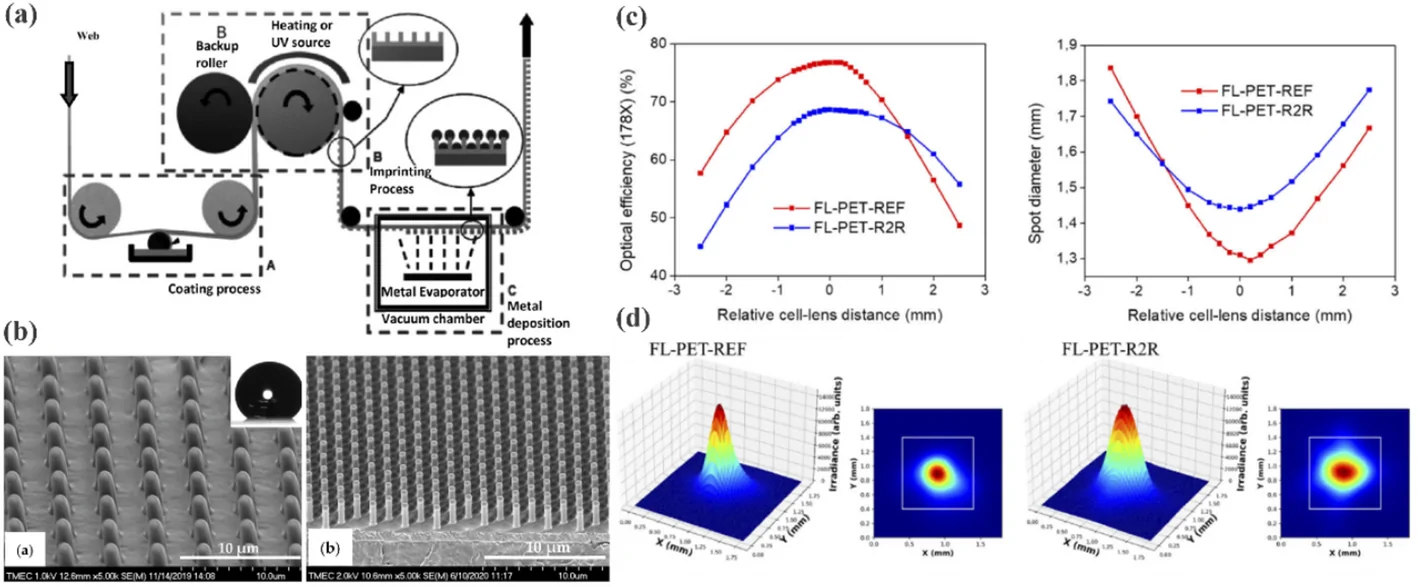
R2R NIL for continuous nanomanufacturing. **a** Line-contact roller imprint configuration. Adapted from Ref [14]. with permission. **b** High-aspect-ratio microstructures fabricated via roll-based imprinting. Adapted from Ref [35]. with permission. **c**–**d** R2R-fabricated Fresnel lens structures and corresponding optical efficiency characterization. Adapted from Ref [37]. with permission

R2R NIL emerged as nanoimprint technology began moving beyond laboratory-scale replication toward continuous industrial manufacturing. With increasing demand for flexible electronics, large-area photonic components, and low-cost energy devices, conventional wafer-based imprinting approaches face limitations in both throughput and substrate size [32, 33]. Roll-based processing provides a practical solution by integrating nanoscale patterning with continuous web manufacturing. Instead of imprinting rigid wafers, R2R NIL performs resist coating, imprinting, curing, and demolding on a moving polymer film within a single continuous process [14]. The basic configuration is illustrated in Fig. 5a, where a patterned roller forms a line-contact interface with the moving substrate. This rolling geometry reduces the imprint force required for pattern transfer and helps suppress defects such as air entrapment and thickness nonuniformity. Because the substrate continuously passes through the imprint region, nanopatterning can be carried out over areas far larger than those accessible with conventional wafer-based systems.

As the technique developed, maintaining structural fidelity during continuous processing became a key challenge. Comparative studies between soft lithography and roll-based imprinting highlight the mechanical advantages of roller-based pattern transfer [33, 34]. The results shown in Fig. 5b illustrate this difference clearly. High-aspect-ratio microstructures fabricated by roll-to-plate nanoimprint lithography exhibit improved structural stability compared with structures produced using PDMS-based soft lithography. Elastomeric molds used in soft lithography may deform or collapse when pattern density becomes high, whereas roller-based imprinting has demonstrated dimensional deviations below 6% for structures with aspect ratios approaching five [35]. These observations suggest that roll-based nanoimprint systems can maintain dimensional accuracy even for mechanically demanding hierarchical nanostructures and large-area functional surfaces.

Uniform pattern replication in continuous R2R systems is also strongly influenced by the fluid dynamics governing resist filling. Recent multiphase modeling studies provide quantitative insight into resin flow behavior under industrial R2R operating conditions [36]. Simulations incorporating sliding-mesh algorithms and volume-of-fluid approaches indicate that imprint speed, resin viscosity, contact angle, and web tension collectively determine filling uniformity and defect formation. Experimental validation confirms that optimized processing conditions can achieve defect-minimized nanopattern replication at industrially relevant web speeds. The capability of R2R NIL has also been demonstrated through device-level fabrication. As shown in Fig. 5c, d, high-efficiency Fresnel lenses have been produced on flexible substrates using R2R UV nanoimprint lithography. Optical efficiencies approaching 69% at concentration ratios of 178× have been reported, closely matching the performance of plate-based reference devices. Integration of moth-eye-inspired antireflective nanostructures through double-sided R2R processing further enhances optical response, showing that continuous roll-based manufacturing can preserve nanoscale optical precision while enabling large-area device production.

Continued development of R2R NIL reflects the broader transition of nanoimprint lithography toward industrial-scale nanomanufacturing. Reviews published after 2010 consistently identify R2R nanoimprint lithography as a promising pathway for scalable nanopattern fabrication [36]. Its advantages include diffraction-unlimited resolution, compatibility with flexible polymer substrates, relatively low capital cost compared with advanced photolithography, and integration with established web-processing infrastructure. Ongoing improvements in mold durability, UV-curable resin chemistry, and tension-controlled web transport are steadily enhancing process reliability and yield stability. These developments indicate that R2R NIL is approaching practical deployment for applications such as flexible photonics, energy harvesting systems, and large-area functional materials. In practical manufacturing, maintaining uniform pattern quality over large areas remains a key challenge for R2R NIL. Variations in web tension can affect imprint uniformity, while roller wear and template degradation may gradually reduce replication fidelity during continuous operation. These factors become increasingly important as the processing area and production volume increase.

### Comparison of NIL techniques and their relative advantages

Nanoimprint lithography comprises several process variants that differ in resist materials, processing conditions, and manufacturing scalability. As summarized in Table 2, the four representative NIL approaches, T-NIL, UV-NIL, S-FIL / J-FIL, and R2R NIL, are optimized for different technological requirements and application scenarios. T-NIL is one of the earliest and most mature NIL techniques. In this approach, thermoplastic polymers are heated above their Tg and deformed under applied pressure to replicate nanoscale mold features [12]. Owing to the deterministic nature of polymer deformation, T-NIL can achieve excellent pattern fidelity and structural accuracy. As a result, this technique has been widely used for nanophotonic gratings, thin-film photovoltaic structures, and microfluidic devices [18, 30]. However, the elevated processing temperature and relatively high imprint pressure may limit throughput and compatibility with temperature-sensitive substrates.

UV-NIL provides an alternative low-temperature patterning route. In UV-NIL, a low-viscosity liquid resist fills nanoscale cavities primarily through capillary forces and is subsequently solidified by ultraviolet exposure at room temperature [38]. Compared with T-NIL, UV-NIL enables lower imprint pressure and shorter cycle times, which improves process efficiency and reduces thermal stress. Consequently, UV-NIL has been widely adopted for optical metasurfaces, glass nanostructures, and various nanophotonic devices where large-area uniformity is required [20, 39]. For semiconductor manufacturing, stepper-based nanoimprint technologies such as S-FIL and J-FIL provide additional advantages. These approaches employ localized step-and-repeat imprinting combined with controlled resist dispensing, allowing precise pattern placement and improved overlay accuracy [9]. The droplet-based resist delivery used in J-FIL further reduces RLT variation and enhances process stability. Such capabilities make stepper-based NIL particularly suitable for semiconductor nanofabrication and integrated circuit applications requiring multilayer alignment and high pattern precision [30]. In contrast to wafer-based imprinting methods, R2R nanoimprint lithography is designed for continuous large-area production. By using flexible substrates transported through roller-based imprint systems, R2R NIL enables high-throughput fabrication of nanostructures over meter-scale areas [33]. This continuous processing architecture significantly improves manufacturing scalability and reduces fabrication cost. As a result, R2R NIL has attracted increasing interest for flexible electronics, optical films, and energy-related devices that require large-area nanostructured surfaces [35, 36].

**Table 2:** Comparison of major nanoimprint lithography techniques and their relative advantages

Comparison parameter	T-NIL	UV-NIL	S-FIL / J-FIL	R2R NIL
Resist type	Thermoplastic polymer	UV-curable liquid resist	UV-curable resist droplets	UV-curable or thermoplastic polymers
Processing temperature	Above Tg	Room temperature	Room temperature	Room temperature or moderate heating
Imprint mechanism	Pressure-driven polymer deformation	Capillary filling followed by UV curing	Drop-on-demand resist dispensing followed by capillary filling and step-and-repeat UV imprinting	Continuous roller-based imprinting
Key advantages	High pattern fidelity and mature process control	Low pressure and short cycle time	High overlay accuracy and compatibility with semiconductor steppers	High throughput and scalable large-area manufacturing
Overlay capability	Limited	Moderate	Excellent (~ 5 nm)	Limited
Manufacturing considerations	Thermal cycling and demolding	Filling uniformity and residual-layer control	Overlay accuracy and defect control	Web tension and defect propagation
Typical applications	Nanophotonic gratings, photovoltaics, microfluidics	Optical metasurfaces, glass nanostructures, photonic devices	Semiconductor nanofabrication and integrated circuits	Flexible electronics, optical films, energy devices
References	[2, 17]	[18, 25]	[29, 39]	[9, 33, 37]

Despite sharing the same fundamental principle of pattern replication through mold-based deformation or curing, the four NIL approaches differ substantially in their processing characteristics and manufacturing capabilities. T-NIL offers excellent pattern fidelity but is constrained by elevated processing temperatures and relatively long cycle times. UV-NIL enables room-temperature fabrication and reduced thermal stress, although filling uniformity and residual-layer control remain important considerations. For applications requiring precise pattern placement and multilayer alignment, S-FIL and J-FIL provide superior overlay performance through localized step-and-repeat imprinting. In contrast, R2R NIL emphasizes continuous large-area production and manufacturing scalability, making it particularly attractive for flexible electronics, optical films, and energy-related devices. Overall, the four NIL techniques provide complementary advantages depending on the targeted application and manufacturing constraints. Thermal NIL offers high pattern fidelity and mature process control, UV-NIL enables low-temperature and high-throughput patterning, stepper-based NIL supports semiconductor-level overlay accuracy, and R2R NIL provides scalable large-area manufacturing capability. These differences highlight that NIL should be considered not as a single lithographic method but as a versatile family of nanofabrication technologies capable of addressing diverse industrial and research needs.

## Materials selection for major nanoimprint lithography techniques

### Template material requirements in different NIL processes

The selection of template materials in nanoimprint lithography is closely related to the operating conditions of each imprint process. Because NIL techniques differ in imprint temperature, pressure, curing mechanism, and process architecture, they impose different mechanical, thermal, optical, and alignment requirements on the mold. Beyond intrinsic material properties, mold selection also affects process-level constraints such as demolding stress, release force, pattern transfer fidelity, defect replication, and template lifetime. These factors are critical because NIL is a contact-based replication process, in which mold deformation, particle contamination, resist residues, and repeated template use can directly influence replication accuracy and defect formation.

#### Thermal NIL: high mechanical strength and thermal stability

T-NIL operates by heating a thermoplastic resist above its Tg while applying significant mechanical pressure to induce viscous flow and cavity filling. Under these conditions, the template is subjected to substantial compressive and bending stresses, making high mechanical stiffness and bending rigidity essential to prevent elastic deformation and dimensional distortion of nanoscale features [1, 2]. Hirai et al. demonstrated that template deformation under imprint pressure directly influences linewidth control and pattern fidelity, particularly for high-aspect-ratio nanostructures [40]. Similarly, Guo emphasized that the mechanical integrity of the mold plays a crucial role in maintaining replication accuracy and dimensional stability during repeated imprint cycles [3]. In addition to mechanical stiffness, thermal stability is a critical requirement for thermal NIL templates. The imprint process involves repeated heating and cooling cycles, which can induce thermo-mechanical stress and thermal expansion mismatch between the template and substrate. Such effects may accumulate over multiple imprint cycles and degrade replication accuracy if the template material does not possess sufficient thermal stability [2, 17]. Consequently, hard materials with high Young’s modulus and good thermal stability, such as silicon and nickel, are commonly used as templates in thermal NIL systems. Because pattern formation in thermal NIL relies on thermoplastic deformation rather than photopolymerization, optical transparency is generally not required for template materials. The high imprint pressure and repeated thermal cycling in T-NIL can increase demolding stress and release force, making thermal stress, release-induced damage, and long-term mold durability the dominant mold-related bottlenecks. These effects may cause feature deformation, mold wear, or particle-induced defect transfer, especially for dense or high-aspect-ratio patterns.

#### UV-NIL and jet-and-flash imprint lithography: optical transparency and surface treatment

Ultraviolet-based nanoimprint lithography techniques, including UV-NIL and J-FIL, operate at significantly lower temperatures and pressures compared with thermal NIL. In these processes, the liquid resist fills the template cavities and is subsequently solidified through UV-induced photopolymerization. As a result, the mechanical stress applied to the template is considerably reduced, and extremely high mechanical rigidity is typically less critical than in thermal NIL. However, optical transparency becomes a key requirement for template materials in UV-based imprint processes. Since ultraviolet radiation must pass through the template to cure the resist while maintaining contact with the substrate, the template material must exhibit high transparency at the curing wavelength. Quartz and fused silica are therefore widely used as template materials due to their excellent UV transmission and dimensional stability [28, 41]. Planarity and overlay capability are also important, particularly for multilayer device fabrication, where accurate alignment between the template and underlying patterns is required. Step-and-repeat UV-NIL systems incorporate alignment architectures to achieve high overlay accuracy across wafer-scale substrates [42]. J-FIL further emphasizes precise alignment between dispensed resist droplets, the template, and the substrate, making overlay control a central consideration in J-FIL process design [5, 43]. Pattern transfer fidelity in UV-NIL and J-FIL is strongly influenced by mold transparency, surface quality, release behavior, and template cleanliness. Incomplete release, particle trapping, or resist residues can generate repeated defects in subsequent imprint fields. In J-FIL, localized droplet filling further increases the need for accurate mold placement and stable field-to-field overlay.

#### R2R NIL: flexible template materials and durability

R2R NIL is designed for large-area, high-throughput nanomanufacturing and therefore introduces different template requirements compared with wafer-based NIL processes. In R2R NIL, the template is typically fabricated as a flexible mold or metallic shim that can conform to the moving substrate during continuous web processing. As a result, extreme mechanical rigidity is not necessarily required, and flexibility becomes advantageous for achieving uniform contact across large areas. The necessity of optical transparency in R2R NIL depends on the curing mechanism employed. In UV-based R2R imprint systems, UV radiation is used to polymerize the resist during continuous web transport, requiring transparent roll molds such as quartz cylinders to allow light transmission. In contrast, thermal roll embossing processes rely on heating to soften the resist and therefore do not require optical transparency [14, 44]. Because the template is used continuously, cumulative wear, contamination, adhesion increase, and repeated demolding forces become major reliability concerns. Increasing adhesion and friction at the mold–resist interface can raise demolding force and accelerate defect formation and template degradation [45]. Unlike wafer-based NIL, defects caused by roller wear, mold contamination, or contact nonuniformity may propagate continuously along the moving web. For soft molds, material degradation, such as resist diffusion into PDMS, can further reduce mold lifetime [46]. Therefore, the dominant mold- and process-related bottlenecks in R2R NIL are roller lifetime, web-tension-induced distortion, mold wear, and continuous defect inspection. Strategies such as anti-adhesion coatings, contamination control, and defect mitigation are therefore important for extending template lifetime and improving manufacturing reliability [16, 47, 48].

Overall, template material requirements in NIL are process-dependent. Thermal NIL emphasizes stiffness and thermal stability; UV-NIL and J-FIL require optical transparency, surface quality, and overlay capability; and R2R NIL prioritizes flexibility, durability, and long-term stability during continuous processing. To reflect both material selection and manufacturing feasibility, Table 3 summarizes the key template requirements, process constraints, representative bottlenecks, and material choices for major NIL techniques.

**Table 3:** Comparison of mold material selection criteria across major NIL techniques

Mold selection criteria	Thermal NIL	UV-NIL / S-FIL	J-FIL	R2R NIL
Mechanical stiffness	High	Moderate	Moderate	Moderate
Thermal stability	High	Low	Low	Moderate
Optical transparency	Low	High	High	Moderate / High (UV or R2R)
Planarity / overlay	Moderate	High	High	Low
Durability / lifetime	Moderate	Moderate	Moderate	High
Demolding stress / release force	High	Moderate	Moderate / High	Moderate / High
Pattern transfer fidelity	Moderate	High	High	Moderate
Defect replication risk	Moderate	Moderate	Moderate / High	High
Key mold-related bottlenecks	Thermal stress / release force	Mold transparency / release failure	Template contamination / overlay	Roller wear / web tension
Representative molds	Si / Quartz / SiC	Quartz / Fused silica	Quartz / Fused silica	Ni / Glassy carbon / PDMS

### Imprint resist requirements in different NIL processes

The selection of imprint resists in NIL is strongly governed by the solidification mechanism and process conditions of each imprint technique. Different NIL methods impose distinct requirements on resist viscosity, filling behavior, curing route, mechanical stability, and throughput compatibility due to variations in imprint temperature, pressure, resist delivery mode, and process architecture. These material requirements are directly linked to key process constraints, including filling time, RLT uniformity, curing shrinkage, demolding stability, and high-throughput compatibility. Thermal NIL, UV-based NIL techniques such as UV-NIL and J-FIL, and R2R NIL, therefore, require different resist properties to ensure complete cavity filling, stable pattern retention, and reliable process performance.

#### Thermal NIL: thermoplastic polymer resists

Thermal NIL employs thermoplastic resists that undergo pattern formation through temperature-induced viscous flow followed by solidification upon cooling. In this process, the resist film is first heated above its Tg to reduce viscosity and enable pressure-driven flow into nanoscale mold cavities during embossing. After cavity filling is achieved, the resist is cooled below Tg while maintaining imprint pressure, which fixes the replicated pattern through thermoplastic solidification rather than chemical crosslinking [17, 49]. Consequently, thermoplastic polymers such as PMMA and related imprint polymers are commonly used in thermal NIL systems. Resist viscosity and flowability play a critical role in determining pattern fidelity in thermal NIL. Because feature filling relies primarily on pressure-driven viscous flow, the polymer must exhibit sufficiently low viscosity at the imprint temperature to ensure complete cavity filling within the available imprint time. At the same time, the resist must retain adequate mechanical stability after cooling to prevent feature deformation or relaxation near Tg. The imprint process window is therefore governed by the combined effects of temperature, pressure, and embossing time, which together determine filling kinetics and replication accuracy [2, 17]. RLT control is also an important consideration in thermal NIL because the residual layer directly affects subsequent etch transfer and critical dimension control. In most thermal imprint processes, RLT is largely determined by the initial resist film thickness and imprint pressure, temperature, and time. Achieving uniform residual layers, therefore, requires well-controlled resist coating and stable process conditions during imprinting [2]. In addition, the thermal cycling inherent to the process introduces cycle-time limitations, since heating and cooling steps must be completed in each imprint cycle. Therefore, the representative resist-related bottlenecks in thermal NIL are slow viscous filling, RLT variation, pattern relaxation near Tg, and throughput limitation caused by repeated thermal cycling.

#### UV-NIL and jet-and-flash imprint lithography: UV-curable low-viscosity resists

UV-NIL and J-FIL employ liquid photocurable resists that solidify through UV-induced crosslinking rather than thermal cooling. In these processes, a low-viscosity liquid resist fills the template cavities while the mold remains in contact with the substrate, and ultraviolet exposure subsequently converts the liquid into a crosslinked polymer network that fixes the imprinted pattern [28, 50]. Because curing occurs through photopolymerization, the imprint process can operate near room temperature and does not require the thermal cycling associated with thermoplastic embossing. Low initial viscosity is an important requirement for UV-based imprint resists because filling occurs primarily through capillary forces rather than pressure-driven polymer flow. Liquid resists with sufficiently low viscosity can rapidly spread and fill nanoscale cavities before UV exposure, thereby minimizing incomplete filling defects and improving pattern uniformity [18, 51]. Acrylate-based UV-curable formulations are therefore widely used due to their fast curing kinetics and tunable mechanical properties. Hybrid organic–inorganic resist systems, such as Ormocer or OrmoComp, have also been developed to provide higher modulus and reduced shrinkage after curing, which improves dimensional stability during subsequent processing [50, 52]. Nevertheless, UV-curable resists must balance fast curing with limited volumetric shrinkage, sufficient etch resistance, and reliable release from the template. Incomplete filling, oxygen inhibition, curing shrinkage, and adhesion-induced deformation can become representative bottlenecks if resist chemistry and UV exposure conditions are not properly optimized. J-FIL further extends this concept by employing field-by-field dispensing of ultra-low viscosity resist droplets using inkjet delivery. The droplets rapidly spread and fill the template features through capillary action before UV exposure, enabling rapid filling kinetics and precise resist volume control [5, 53]. This droplet-based approach also improves RLT control because the dispensed resist volume can be accurately matched to the template cavity volume. After filling is completed, UV curing rapidly solidifies the resist, allowing high overlay accuracy and short cycle times compatible with semiconductor manufacturing requirements. Because pattern fixation occurs through photopolymerization at near room temperature, UV-based NIL processes generally achieve higher throughput than thermal NIL while maintaining high pattern fidelity [28, 53]. For J-FIL, representative resist-related bottlenecks include droplet placement errors, droplet coalescence, air entrapment, and field-to-field RLT variation.

#### R2R NIL: fast-curing and high-throughput compatible resists

R2R NIL is designed for continuous large-area nanomanufacturing and therefore imposes additional constraints on resist materials compared with wafer-based imprint processes. In R2R NIL, imprinting occurs during continuous web transport, so resist materials must support rapid filling, fast solidification, and stable pattern transfer under high-speed processing conditions. The resist formulation used in R2R NIL depends primarily on the curing mechanism implemented in the production line. In UV-assisted R2R imprinting, low-viscosity UV-curable resists are widely used because they can rapidly fill mold features and be solidified through in-line ultraviolet exposure during web transport [14, 54]. Low viscosity is particularly important in this configuration because high web speeds limit the available filling time, requiring rapid spreading and capillary-driven cavity filling across large areas. The curing step is typically integrated directly into the imprint line so that the resist is crosslinked immediately after imprinting, allowing continuous high-throughput operation. Thermal roll embossing processes employ thermoplastic resists that soften upon heating and solidify upon cooling, similar to wafer-based thermal NIL. In such systems, the resist must exhibit appropriate rheological behavior to enable flow under roller pressure while maintaining sufficient mechanical stability after cooling to preserve the replicated features. RLT uniformity is more challenging in R2R NIL because it is influenced by web tension, roller contact mechanics, and resin flow during continuous processing. As a result, resist formulations must be carefully optimized to balance viscosity, curing speed, and mechanical stability in order to achieve consistent pattern transfer across large-area substrates [14, 55, 56]. Representative bottlenecks include insufficient filling at high web speed, premature or incomplete curing, RLT nonuniformity across the moving web, and material-dependent degradation during continuous operation.

Overall, resist selection in NIL is strongly influenced by the solidification mechanism and process architecture of each NIL technique. Thermal NIL relies on thermoplastic polymers whose viscosity and flow behavior determine cavity filling during embossing. UV-based NIL techniques, including UV-NIL and J-FIL, employ photocurable liquid resists designed for rapid capillary filling and UV crosslinking at near room temperature. In R2R NIL systems, resist materials must additionally support continuous processing conditions, requiring rapid filling kinetics, fast curing or cooling, and stable pattern transfer during high-speed web transport. To reflect both material selection and manufacturing feasibility, the key resist requirements, process constraints, representative bottlenecks, and material systems for different NIL techniques are summarized in Table 4.

**Table 4:** Comparison of imprint resist requirements across different NIL techniques

Resist selection criteria	T-NIL	UV-NIL / S-FIL	J-FIL	R2R NIL
Resist type	Thermoplastic polymer	UV-curable liquid resist	UV-curable droplet-based resist	UV-curable or thermoplastic resist
Initial viscosity/flowability	High viscosity at room temperature; softened above Tg	Low viscosity liquid	Ultra-low viscosity liquid	Low viscosity (for rapid filling)
Filling mechanism	Thermally induced viscous flow under pressure	Capillary-driven filling	Drop-wise spreading followed by capillary flow	Continuous contact filling
Curing / solidification mechanism	Cooling below Tg	UV-induced crosslinking	UV-induced crosslinking	UV curing or thermal cooling
Process temperature	Elevated temperature (typically 100–200 °C)	Room temperature	Room temperature	Room temperature to moderate temperature
RLT control	Moderate; dependent on pressure, temperature, and resist thickness	Good; controlled by resist volume and imprint gap	Excellent; precisely defined by dispensed droplet volume	Challenging over large areas
Mechanical stability after imprint	High after cooling and solidification	High after crosslinking	High after crosslinking	Moderate to high (material-dependent)
Throughput compatibility	Limited by heating and cooling cycles	High	High; suitable for wafer-scale patterning	Very high; compatible with continuous processing
Key process constraints	Temperature-pressure-time window; thermal cycling	Capillary filling, UV dose, shrinkage, and release control	Droplet placement, spreading, overlay, and volume control	Web speed, web tension, roller contact, and in-line curing/cooling
Representative resist materials	PMMA, PS, thermoplastic imprint polymers	Acrylate-based UV resists	Low-viscosity acrylate-based UV resists	UV-curable acrylates, polyurethane acrylates

### Substrate compatibility in different NIL processes

The selection of substrates in NIL is strongly governed by the mechanical, thermal, and geometric demands of each imprint technique. Different NIL methods impose distinct requirements on substrate rigidity, thermal tolerance, surface planarity, dimensional stability, and chemical compatibility due to variations in imprint temperature, pressure, alignment accuracy, and process architecture. These substrate requirements are directly associated with key process constraints, including contact uniformity, thermal deformation, overlay stability, surface wettability, and large-area handling reliability. Thermal NIL, UV-based NIL techniques such as UV-NIL and J-FIL, and R2R NIL therefore require different substrate properties to ensure stable contact, reliable pattern transfer, and consistent process performance.

#### T-NIL: rigid substrates with high thermal stability

Thermal NIL typically requires substrates that can tolerate elevated temperature and pressure during the imprint process. Because pattern transfer occurs by heating a thermoplastic resist above its Tg and subsequently cooling below Tg, the substrate must maintain dimensional stability throughout repeated thermal cycles. Materials with high thermal tolerance, such as silicon, glass, and sapphire, are therefore commonly employed, as they resist warpage and deformation during heating and cooling [17]. Thermal expansion mismatch between the substrate and imprint stack can also introduce in-plane strain and pattern distortion during cooling, making thermal stability an important consideration in substrate selection [3, 17]. Mechanical rigidity is another key requirement for substrates used in thermal NIL. Rigid substrates help maintain a stable contact geometry between the mold and the resist layer, preventing local gap variations that may lead to incomplete cavity filling or non-uniform pattern transfer. Substrate bow or waviness can become amplified under imprint pressure and thermal cycling, potentially leading to RLT variation and defect formation across the patterned area. As a result, rigid and planar substrates are generally preferred to ensure uniform pressure distribution and reliable replication fidelity. Surface chemistry also influences resist adhesion and wetting behavior during imprinting. Poor interfacial compatibility may lead to resist dewetting, trapped voids, or delamination during demolding. Consequently, surface treatments such as oxygen plasma activation are often used to increase surface energy and improve resist wetting and adhesion on silicon or oxide substrates. Therefore, the representative substrate-related bottlenecks in T-NIL are thermal expansion mismatch, substrate warpage, pressure-induced RLT variation, and delamination or defect formation during demolding. These considerations collectively make rigid, thermally stable substrates the preferred choice for thermal NIL processes.

####  UV-NIL and jet-and-flash imprint lithography: transparent and surface-modified substrates

UV-NIL and J-FIL typically operate near room temperature and therefore impose less stringent thermal requirements on the substrate compared with thermal NIL. Since pattern fixation occurs through ultraviolet-induced crosslinking rather than thermal cooling, the substrate does not need to withstand large temperature swings during the imprint process. Instead, substrate selection is more strongly influenced by surface chemistry, dimensional stability, and compatibility with the resist and lithographic alignment system [3, 5]. Rigid substrates such as silicon and glass are widely used in wafer-based UV-NIL systems because they provide excellent mechanical stability and surface planarity. High substrate flatness enables uniform contact between the template and the resist layer, which is essential for achieving consistent cavity filling and low defect density across large patterned areas. Planarity is particularly important in step-and-repeat UV-NIL processes, where accurate alignment between successive imprint fields is required to maintain overlay accuracy across the wafer [42]. In J-FIL, substrate dimensional stability becomes even more critical because the process targets advanced semiconductor manufacturing. J-FIL is commonly implemented on silicon wafers in stepper-style tool architectures, where precise alignment between dispensed resist droplets, the template, and underlying device layers is required to achieve nanometer-scale overlay accuracy [5, 54]. Surface energy and wettability also influence droplet spreading and resist distribution before UV curing, meaning that substrate surface preparation plays an important role in ensuring reliable pattern transfer and process stability. The main substrate-related constraints for UV-NIL and J-FIL are substrate flatness, surface-energy control, dimensional stability, and compatibility with overlay alignment. If these factors are not properly controlled, representative bottlenecks include local contact nonuniformity, void formation, droplet spreading variation, overlay error, and field-to-field RLT variation.

#### R2R NIL: flexible substrate compatibility

R2R NIL employs flexible substrates in the form of continuous polymer webs, enabling large area and high throughput nanomanufacturing. Unlike wafer-based NIL techniques, R2R NIL typically uses mechanically compliant substrates such as PET, PEN, PI, and other flexible polymer films. The flexibility of these substrates allows conformal contact with the roller mold during continuous web transport, facilitating uniform pattern transfer across large areas [14, 54]. Because R2R processes often operate at high web speeds, substrate mechanical behavior becomes closely coupled to web handling conditions. Parameters such as web tension, bending stiffness, and deformation under roller pressure can influence contact mechanics during imprinting and therefore affect filling uniformity and RLT across the patterned region [55]. Maintaining stable web transport and minimizing mechanical distortion are therefore essential for achieving consistent pattern replication in continuous processing. Thermal tolerance requirements in R2R NIL depend on the curing mechanism used in the imprint line. In UV-assisted R2R processes, ultraviolet curing allows pattern fixation at relatively low temperatures, which is compatible with many polymer films and enables high-speed continuous processing. In contrast, thermally assisted roll embossing requires substrates that can tolerate heating without excessive shrinkage or dimensional instability. As a result, substrate materials used in R2R NIL must be carefully selected to balance flexibility, dimensional stability, and compatibility with high-throughput web-based manufacturing environments. Representative bottlenecks in R2R NIL include web-tension-induced distortion, substrate stretching or shrinkage, roller-pressure-induced nonuniformity, and handling defects during continuous transport.

Overall, substrate compatibility in NIL is largely determined by the mechanical, thermal, and geometric constraints of each NIL process. Thermal NIL typically requires rigid substrates with high thermal stability to withstand elevated temperature and pressure during embossing. UV-based NIL techniques, including UV-NIL and J-FIL, operate near room temperature and therefore emphasize substrate flatness, surface chemistry, and dimensional stability for reliable alignment and pattern transfer. In R2R NIL systems, flexible polymer webs enable continuous large-area processing, making web mechanics, thermal tolerance, and handling stability key factors for uniform high-throughput imprinting. To reflect both substrate selection and manufacturing feasibility, the representative substrate materials, key process constraints, and substrate-related bottlenecks for different NIL techniques are summarized in Table 5.

**Table 5:** Substrate requirements and compatibility across different NIL techniques

Substrate selection criteria	T-NIL	UV-NIL / S-FIL	J-FIL	R2R NIL
Thermal tolerance	High	Low	Low	Low to moderate
Mechanical rigidity	High	High	High	flexible
Surface flatness / planarity	Moderate	High	Very high	Low to moderate
Thermal expansion mismatch sensitivity	High	Low	Low	Moderate
Chemical compatibility with resist	High temperature stable	UV compatible	UV compatible	UV / durable
Dimensional stability	High	High	Very high	Moderate
Large-area scalability	Limited	Moderate	Limited	Excellent
Key process constraints	Thermal cycling, pressure uniformity, substrate warpage	Contact uniformity and surface wetting	Flatness, overlay accuracy, and droplet spreading	Web tension, roller contact, and transport stability
Representative substrates	Si, glass, sapphire	Si, glass, quartz	Si wafers	PET, PEN, PI

## Key application domains

### Nanophotonic and meta-optical applications of NIL

**Fig. 6:**
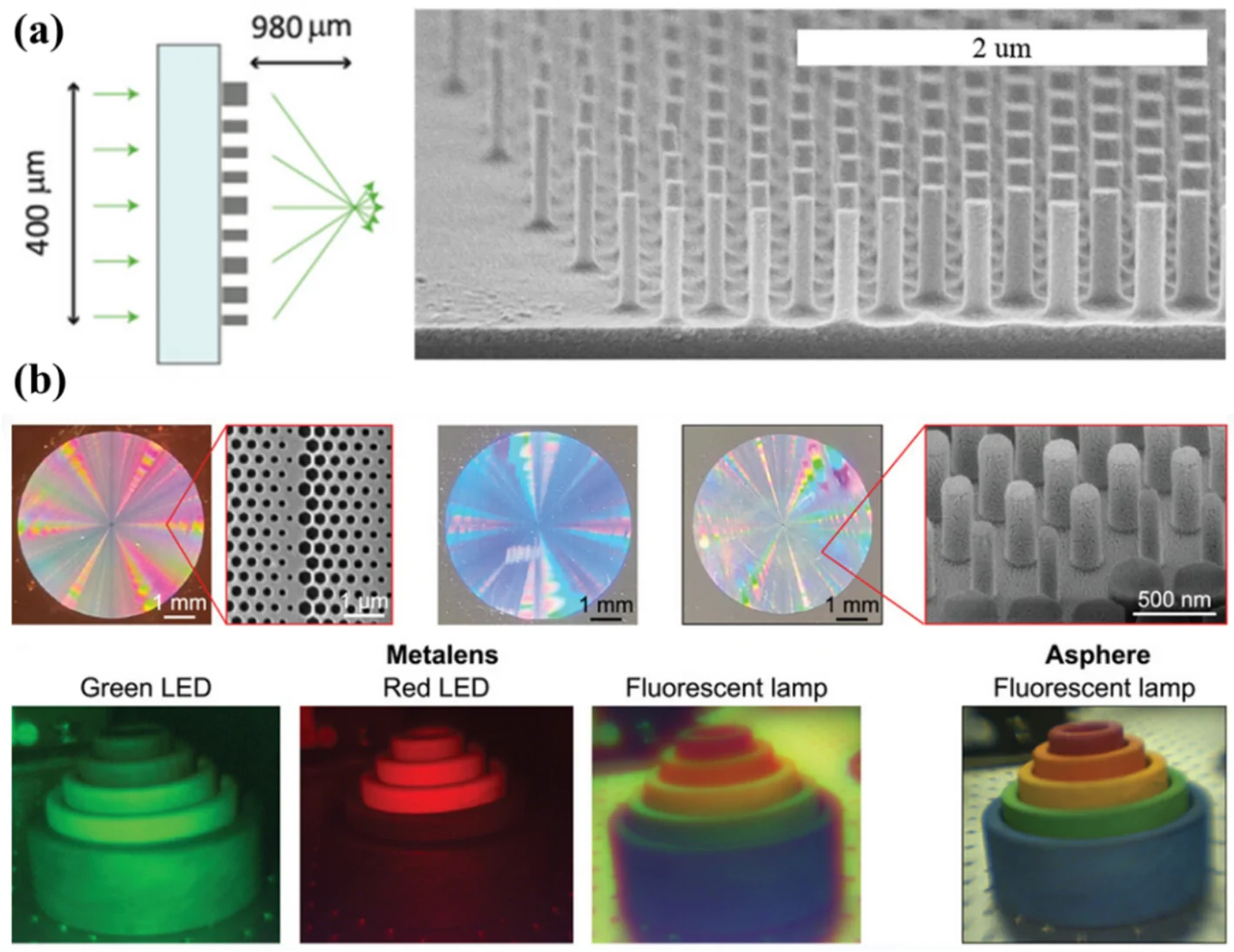
NIL-lithography-enabled meta-optical devices. **a** Schematic and SEM image of a visible metalens composed of high-aspect-ratio TiO_2_ meta-atoms. Adapted from Ref [66]. with permission. **b** Large-area nanoimprinted metalenses showing uniform replication and effective focusing under LED and broadband illumination. Adapted from Ref [67]. with permission

Among the various application domains of NIL, nanophotonic and meta-optical devices represent one of the most compelling use cases because device performance is directly determined by nanoscale structural geometry. Visible-wavelength metasurfaces typically require sub-100 nm feature sizes, high aspect ratios, and wafer-scale uniformity, making large-area fabrication challenging for conventional lithography techniques. NIL is particularly attractive in this field because it enables deterministic replication of high-resolution meta-atoms over large areas while significantly reducing fabrication time compared with serial electron-beam lithography. Nanophotonic and meta-optical devices rely on precisely engineered subwavelength structures to manipulate the phase, amplitude, and polarization of light with spatial resolutions well below the operating wavelength. These concepts have enabled a new generation of flat optical components, including metalenses [57, 58], beam-shaping elements [59, 60], and gradient-index antireflection (AR) surfaces [61, 62]. However, the manufacturability of such devices remains a critical challenge, as visible-wavelength metasurfaces typically require feature sizes below 100 nm and aspect ratios exceeding 5, which are difficult to achieve with conventional photolithography and prohibitively slow to fabricate by EBL [63, 64]. Early efforts to apply NIL to nanophotonic structures primarily focused on polymer-based or low-index materials, which limited optical efficiency [65]. A major breakthrough was reported in 2021 by Einck et al. (Watkins and Arbabi groups), who demonstrated a direct-imprint NIL process for visible metalenses composed of high-aspect-ratio TiO_2_ meta-atoms, as shown in Fig. 6a [66]. In that work, TiO_2_ nanocrystal-based inks were used to fabricate nanoposts with critical dimensions below 60 nm and aspect ratios up to 8.4, yielding metalenses operating at 550 nm with numerical apertures of NA ≈ 0.2 and measured focusing efficiencies exceeding 50%. Importantly, the authors demonstrated 15 consecutive imprints using a single stamp within 30 min, with negligible degradation in optical performance, highlighting the intrinsic scalability of NIL for metasurface manufacturing. Building on this foundation, subsequent studies have focused on improving efficiency and compatibility with established dielectric platforms. In 2024, McClung et al. reported a mask-templating NIL approach for fabricating visible silicon nitride (SiN) metalenses [67]. Using composite PDMS stamps and conventional dry etching, the group realized 6 mm-diameter metalenses operating at 550 nm with numerical apertures of 0.2 and peak focusing efficiencies of 81%, approaching the performance of SiN-fabricated reference devices (89%). Figure 6b depicts imprinted nano-posts with widths ranging from 100 to 310 nm and heights of approximately 650 nm, demonstrating that NIL can achieve the dimensional precision required for high-efficiency propagation-phase metasurfaces while enabling wafer-scale replication.

**Fig. 7:**
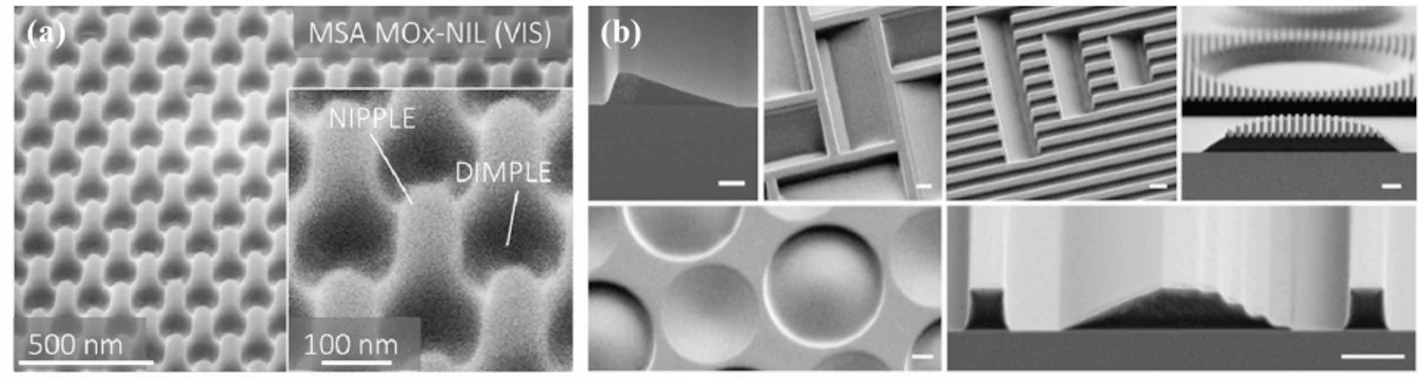
NIL-enabled advanced nanophotonic surfaces. **a** MO_x_-NIL-fabricated nipple-dimple antireflective nanostructures with broadband and wide-angle response. Adapted from Ref [68]. with permission. **b** Quasi-continuous nano-optical topographies formed by thermal reflow of NIL-patterned polymers. (Scale bars: 1 μm) Adapted from Ref [69]. with permission

In addition to metalenses, NIL has enabled nanophotonic surfaces with broadband, angularly robust optical responses. In 2024, Modaresialam et al. reported nanoimprinted metal-oxide antireflective structures with three-dimensional nipple-dimple architectures, fabricated via sol-gel-based MO_x_-NIL, as shown in Fig. 7a [68]. These structures achieved total transmission exceeding 99.5% from 390 to 900 nm and maintained transmission above 99% for incident angles up to 50°. Notably, the reported laser-induced damage thresholds exceeded 150 J/cm^2^ (12 ns, 1064 nm) and 5 J/cm^2^ (500 fs, 1030 nm), approaching those of bulk fused silica substrates. Such results underscore NIL’s capability to produce mechanically robust, high-performance nanophotonic surfaces suitable for demanding optical environments, including high-power laser systems. In parallel, NIL has been extended toward the fabrication of non-binary and quasi-continuous meta-optical topographies, which offer additional degrees of freedom compared to discrete meta-atom arrays. Earlier foundational work on thermally assisted topography equilibration, reported by Schleunitz et al. in 2014, demonstrated that polymer structures patterned by NIL could be selectively reflowed to form smooth convex, concave, and sloped nanoscale profiles with surface roughness on the order of a few nanometers, as shown in Fig. 7b [69]. Although originally explored for polymer optics and microfluidics, such approaches have since influenced the development of advanced NIL-based strategies for creating gradient and hybrid nanophotonic structures that bridge the gap between binary metasurfaces and continuous freeform optics.

Taken together, these results illustrate a clear evolution of NIL-enabled nanophotonics over the past decade, from early polymer-based demonstrations to high-efficiency, visible-wavelength dielectric metasurfaces and robust three-dimensional optical nanostructures. By consistently achieving sub-100 nm feature sizes, millimeter-scale apertures, and wafer-level reproducibility, NIL serves as a highly scalable manufacturing pathway for translating meta-optical concepts from laboratory-scale prototypes to practical photonic systems [16].

### Integrated and silicon photonic applications of NIL

Integrated silicon photonic devices impose a different set of fabrication requirements compared with metasurfaces. In addition to dimensional accuracy, optical loss is highly sensitive to sidewall roughness and pattern fidelity. Therefore, the key value of NIL in silicon photonics is not merely its high resolution, but its ability to reproduce smooth and uniform nanostructures over large wafer areas while maintaining low optical scattering losses [70, 71]. Silicon photonic integrated circuits (PICs) typically require periodic or subwavelength structures with feature widths ranging from several hundred nanometers down to below 100 nm, together with tight duty-cycle control and smooth sidewall profiles. A primary focus in this domain is the fabrication of high-aspect-ratio optical waveguides and high-Q microring resonators (MRR). Because the performance of these devices is highly sensitive to sidewall roughness, NIL must achieve extreme fidelity during pattern transfer.

**Fig. 8:**
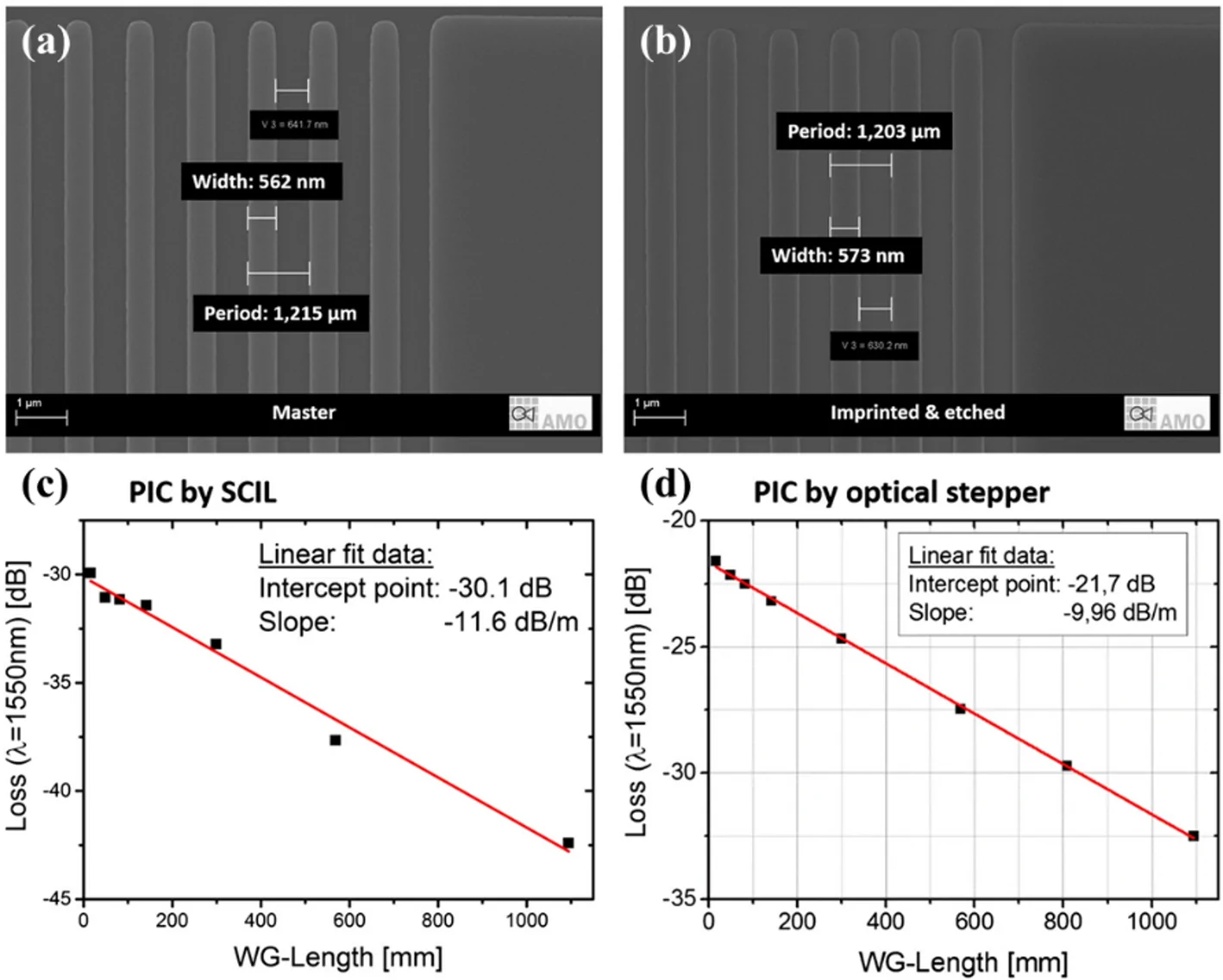
NIL-fabricated silicon photonic waveguides and loss comparison. **a** Master grating structure. **b** Imprinted and etched waveguide. **c** Propagation loss of SCIL-fabricated PIC (11.6 dB/m at 1550 nm). **d** Reference PIC fabricated by optical stepper (9.96 dB/m). Adapted from Ref [72]. with permission

A representative example is shown in Fig. 8a, b, where submicron waveguide gratings fabricated by SCIL are compared before and after imprinting and etching [72]. The master structure exhibits a period of approximately 1.215 μm and a linewidth of 562 nm, while the imprinted and etched structure maintains a period of 1.203 μm and a linewidth of 573 nm, indicating dimensional deviations below ~ 2%. Such dimensional fidelity confirms that NIL can accurately replicate silicon photonic patterns across large areas. More importantly, device-level optical performance further validates the suitability of NIL for integrated photonics. As illustrated in Fig. 8c, d, the propagation losses of waveguides at a wavelength of 1550 nm were determined from PICs fabricated via SCIL and subsequently compared with those obtained from devices manufactured using a conventional optical stepper [72]. The NIL-defined PIC exhibited a propagation loss of approximately 11.6 dB/m, while the stepper-fabricated reference showed 9.96 dB/m. Although slightly higher, the NIL result remains within the same order of magnitude, demonstrating that imprint-induced line-edge roughness and sidewall scattering can be controlled to levels compatible with telecom-grade silicon photonics. This comparison highlights that NIL can achieve performance close to advanced optical lithography while offering parallel processing and potentially lower cost per wafer.

**Fig. 9:**
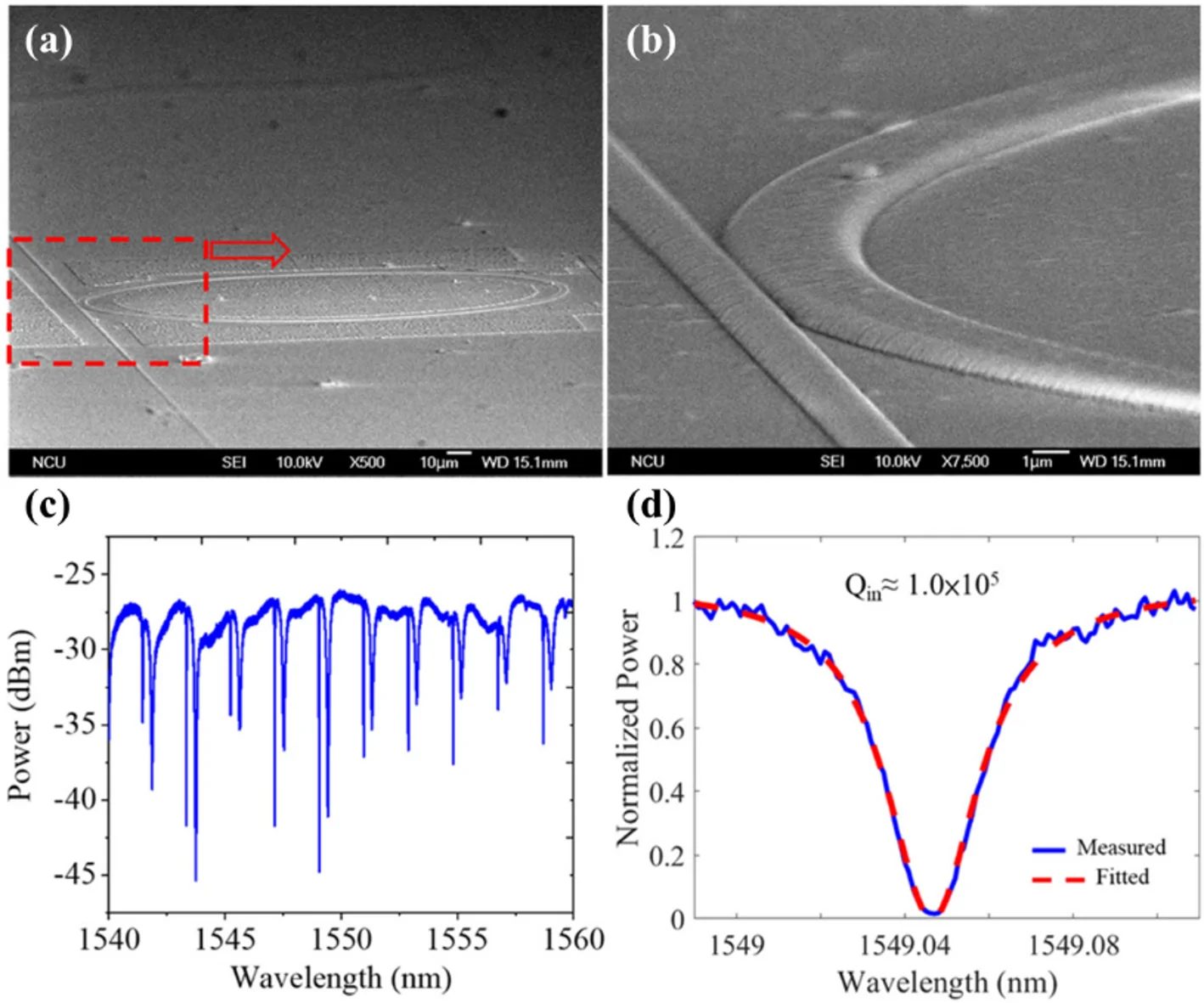
NIL-defined microring resonator on a silicon photonics platform. **a**, **b** SEM images of the imprint-defined ring structure. **c** Measured transmission spectrum. **d** Fitted resonance profile. Adapted from Ref [71]. with permission

In addition to linear waveguide structures, NIL has been successfully applied to resonant photonic components. Figure 9 presents SEM images of an imprint-defined microring resonator fabricated on a silicon photonics platform [71]. The well-defined curved waveguide profiles and smooth sidewalls confirm the capability of NIL to reproduce complex, non-linear geometries required for resonant devices. Optical characterization reveals multiple resonance dips in the 1540–1560 nm wavelength range, with an intrinsic quality factor Q_in_ approaching 1.0 × 10^5^. Such Q factors indicate low optical loss and minimal fabrication-induced roughness, underscoring that NIL is not limited to periodic gratings but can support high-Q integrated resonators suitable for filtering, modulation, and sensing applications.

The above studies indicate that NIL has evolved from an experimental nanofabrication technique to a practical approach for fabricating silicon photonic components, including low-loss waveguides, periodic gratings, and high-Q resonators [16, 70, 73]. The ability to maintain submicron dimensional accuracy, meter-scale-equivalent propagation losses below ~ 12 dB/m, and intrinsic quality factors on the order of 10^5^ establishes NIL as a technically credible fabrication strategy for large-scale photonic integration. More recently, NIL has gained renewed attention in the context of optical interconnects and co-packaged optics (CPO) for data-center applications [70, 74]. As electrical interconnect bandwidth approaches fundamental limits, silicon photonics has emerged as a key enabler for high-speed optical I/O. In this landscape, NIL offers a scalable pathway to replicate dense arrays of grating couplers, microlenses, and alignment features across full wafers with high repeatability, potentially reducing the manufacturing cost of high-volume optical transceivers. By combining sub-100 nm resolution, wafer-scale throughput, and compatibility with established semiconductor materials such as Si and SiN, NIL is transitioning from a pattern-replication technique for discrete photonic components to a viable manufacturing platform for integrated silicon photonics. As photonic integrated circuits expand into data communication, sensing, and AI-driven optical interconnect systems, nanoimprint lithography is increasingly positioned as a cost-effective and manufacturable solution for high-density photonic integration. Despite these promising demonstrations, a remaining technical bottleneck for mass-producing telecom-grade PICs via NIL lies in the precise control of the RLT uniformity across large-area substrates. Any localized variation in the RLT can introduce critical dimension (CD) deviations during the subsequent reactive-ion etching (RIE) process, altering the targeted effective refractive index and potentially degrading device yield.

### Optoelectronic and energy-related applications of NIL

In optoelectronic and energy-related devices, the primary advantage of NIL lies in its ability to engineer light–matter interactions through large-area nanoscale surface structures. Unlike semiconductor manufacturing, where overlay accuracy is often the dominant concern, LEDs and photovoltaic devices primarily benefit from enhanced light extraction, reduced optical reflection, improved light trapping, and more efficient photon management. Consequently, NIL has emerged as an effective fabrication strategy for improving device performance without fundamentally altering the underlying active material system [10, 41, 75, 76].

**Fig. 10:**
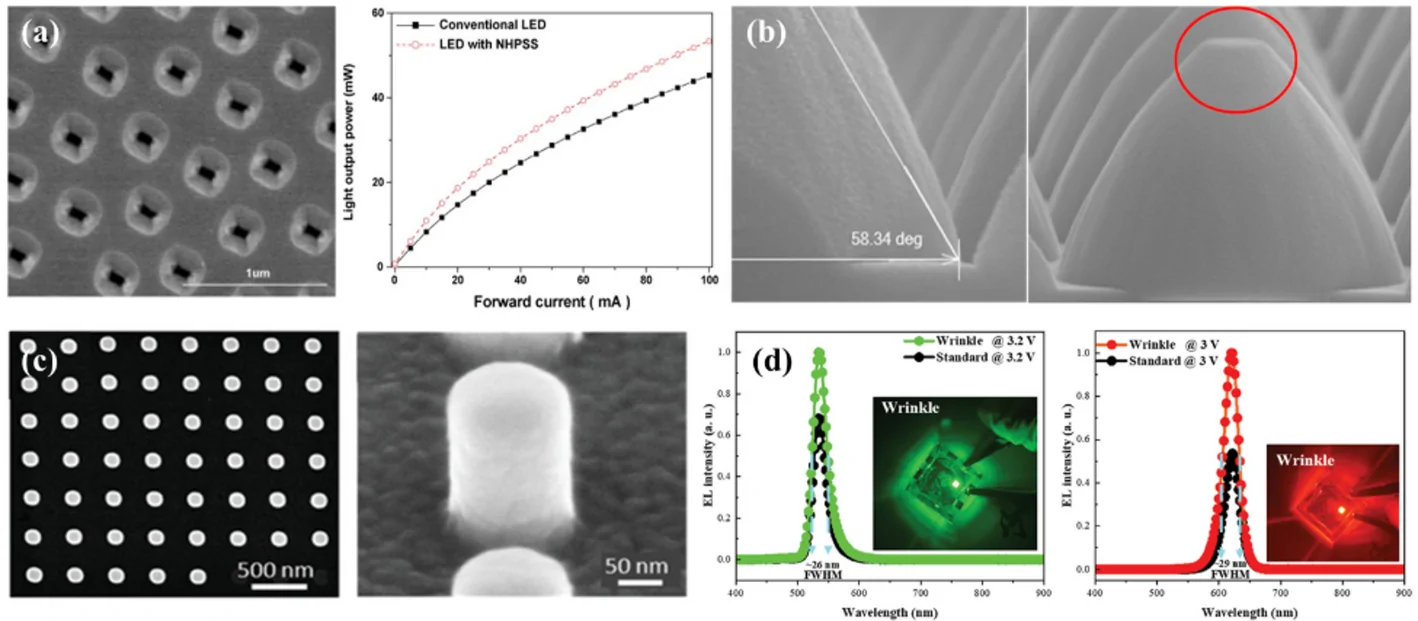
NIL-enabled nanostructured GaN-based LED. **a** Periodic nanohole structures fabricated on GaN LED surfaces using NIL and the corresponding light output power comparison between conventional LEDs and nano-patterned devices. Adapted from Ref [78]. with permission. **b** Cross-sectional SEM showing well-defined sidewalls. Adapted from Ref [16]. with permission. **c** Uniform nanopillar arrays with submicron pitch. Adapted from Ref [79]. with permission. **d** EL spectra of NIL-patterned wrinkle QLEDs and standard QLEDs, showing enhanced EL intensity through improved light out-coupling and carrier injection. Adapted from Ref [80]. with permission

In light-emitting devices, particularly GaN-based visible LEDs and emerging micro-LED architectures, surface nanostructuring plays a crucial role in overcoming total internal reflection at the semiconductor-air interface. Hirai and co-workers demonstrated that UV-NIL fabrication enables the transfer of periodic nanostructures with submicron pitch onto sapphire or GaN surfaces via controlled imprinting and reactive-ion etching [77]. The resulting features typically exhibit lateral dimensions of 200–500 nm, providing effective diffraction-assisted outcoupling at visible wavelengths. Figure 10a shows NIL-defined periodic nanostructures integrated on GaN-based LEDs. The submicron nanohole patterns modify the semiconductor-air interface and promote diffraction-assisted photon extraction. Consequently, the nano-patterned LEDs deliver an output power of ~ 50 mW at 100 mA, compared with ~ 42–45 mW for conventional flat devices, corresponding to an improvement of approximately 15–20% [78]. Figure 10b presents the cross-sectional morphology of the nanoimprinted structures, revealing well-defined sidewall angles of approximately 58° and smooth top surfaces [16]. Such geometrical control is critical for minimizing scattering loss while maintaining strong optical extraction. The replicated structures exhibit dimensional deviations of less than a few percent relative to the master, confirming high-fidelity transfer. The top-view SEM images in Fig. 10c further illustrate uniform nanopillar arrays with feature diameters of ~ 300–500 nm and periodic spacing in the submicron regime [79]. The structural uniformity across the patterned region ensures consistent angular emission characteristics over large LED areas, which is particularly important for micro-LED display arrays. Collectively, the results presented in Fig. 10a–c demonstrate that NIL-enabled surface nanostructuring can effectively improve photon extraction and emission control in GaN-based LEDs through deterministic nanoscale pattern replication.

Beyond GaN-based LEDs, NIL has also been explored in other electroluminescent device platforms. Zhao et al. demonstrated wrinkle-assisted NIL patterning of PEDOT: PSS layers in quantum-dot light-emitting diodes (QLEDs), where the nanoimprinted structures simultaneously enhanced light out-coupling and carrier injection efficiency [80]. As shown in Fig. 10d, the NIL-treated wrinkle QLEDs exhibit noticeably higher electroluminescence (EL) intensity than standard devices operated at comparable driving voltages. While the emission wavelengths remain essentially unchanged, the enhanced EL intensity indicates improved optical extraction and charge transport resulting from the NIL-induced surface morphology and molecular orientation effects. These findings suggest that the benefits of NIL are not limited to GaN-based LEDs but can also be extended to emerging QLED architectures through both optical and electrical performance enhancement mechanisms. Overall, these demonstrations highlight the versatility of nanoimprint lithography as a scalable nanofabrication platform for light-emitting devices. By enabling precise nanoscale structural control, NIL can enhance light extraction, emission characteristics, and device efficiency across a wide range of optoelectronic technologies, including both GaN-based LEDs and QLEDs.

**Fig. 11:**
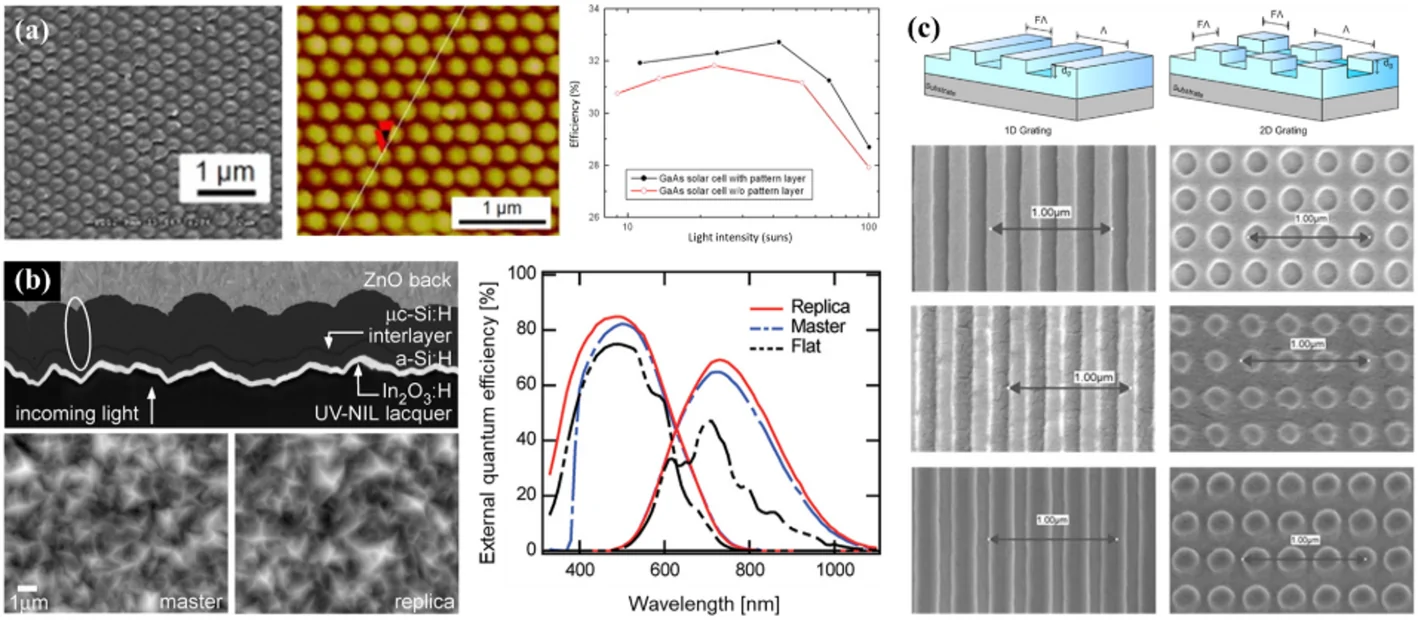
NIL-enabled nanostructures for photovoltaic enhancement. **a** Nanoimprinted dome textures on GaAs solar cells showing enhanced photocurrent. Adapted from Ref [81]. with permission. **b** UV-NIL-patterned thin-film silicon solar cells with broadband absorption improvement. Adapted from Ref [82]. with permission. **c** One- and two-dimensional grating structures for diffraction-assisted light trapping. Adapted from Ref [23]. with permission

In photovoltaic and energy-harvesting devices, NIL has been extensively explored as a scalable approach for light trapping and broadband antireflection. Subwavelength dome arrays, quasi-random textures, and periodic grating structures fabricated by UV-NIL or thermal NIL have been applied to silicon, GaAs, and thin-film solar cells to enhance optical path length and reduce reflection losses. As shown in Fig. 11a, b, nanoimprinted surface textures integrated into thin-film solar cell stacks increase optical absorption across the visible spectrum compared with unpatterned thin-film solar cell stacks [81, 82]. For example, nanoimprinted dome-type textures on GaAs solar cells have been reported to increase short-circuit current density (J_sc_) by approximately 5–10%, with the most pronounced enhancement occurring in the 400–600 nm wavelength range where front-surface reflection is dominant. Reflectance reduction from ~ 30% to below 10% in parts of the visible spectrum has also been observed, depending on texture geometry. Similarly, replicated nanostructures in µc-Si: H and a-Si: H thin-film cells exhibit absorption spectra that closely match the master patterns, with broadband absorption enhancement exceeding ~ 15% in the 400–800 nm range compared to flat references. Optical measurements comparing master, replica, and flat devices confirm that NIL replication maintains the light-trapping functionality of the original texture while enabling scalable fabrication. In several thin-film configurations, these enhancements translate into overall power conversion efficiency improvements on the order of 1–2% (absolute), depending on absorber thickness and back-reflector design. In addition to quasi-random textures, NIL enables precise one- and two-dimensional grating geometries for diffraction-assisted light trapping, as illustrated in Fig. 11c [23]. By controlling grating period (typically 500–1000 nm), fill factor, and depth, such structures enhance coupling into guided modes within thin absorber layers, thereby increasing optical path length without increasing material thickness. Experimental studies have shown absorption enhancement of up to ~ 20% at specific resonance wavelengths compared with flat devices. The ability to fabricate these geometries uniformly over large areas makes NIL compatible with scalable photovoltaic manufacturing. Importantly, NIL-defined metal-oxide or polymer-based surface layers can be integrated without significantly altering underlying semiconductor processing flows, preserving electrical characteristics while improving optical efficiency.

Overall, these demonstrations show that nanoimprint lithography enables deterministic nanoscale patterning for photovoltaic devices, resulting in enhanced light trapping, increased short-circuit current density, improved optical absorption, and higher power conversion efficiency. These improvements demonstrate the potential of NIL as a scalable fabrication technology for next-generation photovoltaic and energy-harvesting devices.

### Large-area and flexible photonic applications of NIL

Large-area and flexible photonics represent an application domain where the core selection logic for NIL shifts toward macroscale area scalability, continuous high-throughput replication, and mechanical substrate conformity, attributes that are inaccessible via conventional wafer-bound optical steppers [75, 83–85]. Unlike serial lithographic techniques, NIL enables parallel pattern transfer across centimeter- to wafer-scale substrates in a single imprint step, making it particularly suitable for large-area metasurfaces, photonic crystal slabs, and structured light-management layers.

**Fig. 12:**
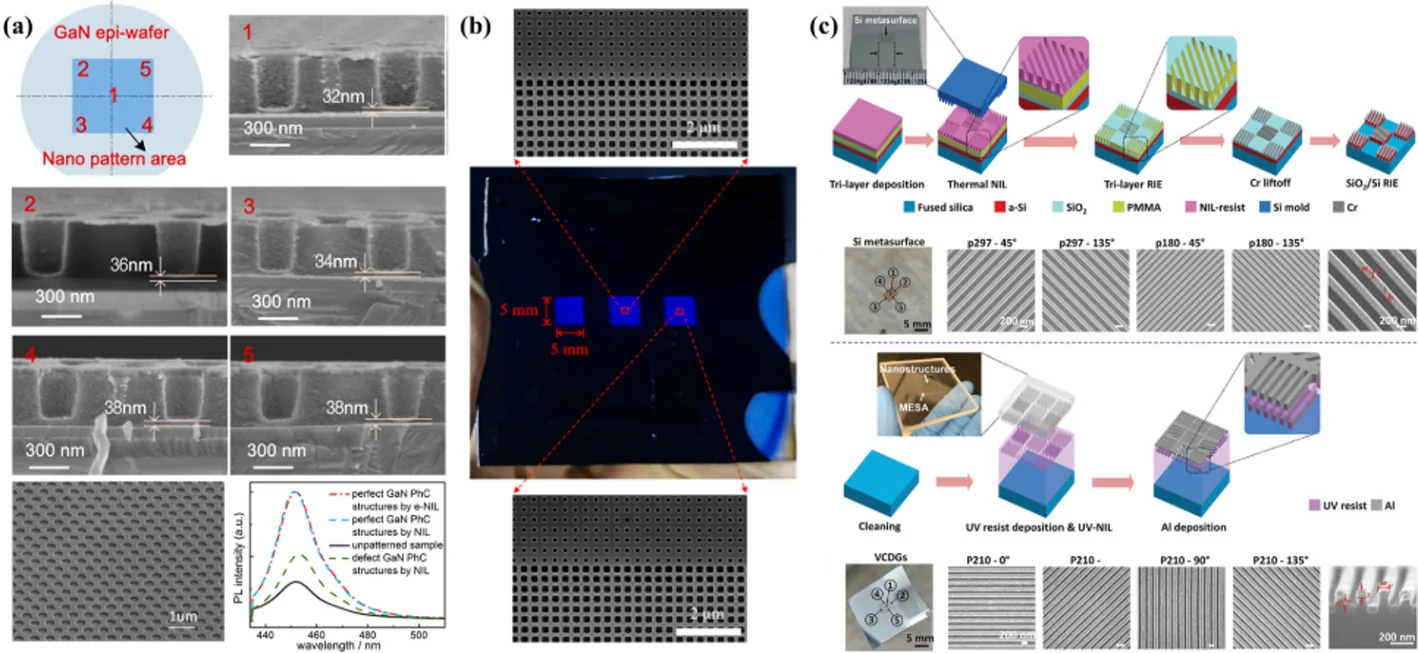
Large-area NIL-fabricated photonic nanostructures on rigid substrates. **a** Wafer-scale photonic crystal nanopatterns replicated on a 4-inch GaN LED epi-wafer using electric-field-assisted NIL. Adapted from Ref [86]. with permission. **b** High-uniformity nanohole/nanopillar arrays over millimeter-scale areas. Adapted from Ref [42]. with permission. **c** Centimeter-scale metasurface device demonstrating scalable pattern replication. Adapted from Ref [85]. with permission

As shown in Fig. 12a, wafer-scale nanoimprint lithography has been demonstrated for large-area nanopatterning on semiconductor substrates. Using an electric-field-assisted NIL process, photonic crystal nanostructures (~ 300 nm feature size) were replicated across a 4-inch GaN LED epi-wafer. The flexible template enables conformal contact with slightly curved substrates (bow ~ 158 μm), while cross-sectional analysis shows a uniform residual resist layer with thickness variations below ~ 8 nm, confirming the scalability of NIL for large-area photonic device fabrication [86]. Uniform nanopattern arrays with pitches of ~ 280 nm and feature heights around 300–350 nm have been replicated with high fidelity, demonstrating scalability beyond small test chips. In Fig. 12b, dense periodic nanostructures fabricated on silicon exhibit consistent critical dimensions across millimeter-scale fields, confirming defect densities sufficiently low for photonic device applications [42]. Such dimensional control is crucial for preserving phase accuracy and diffraction efficiency in large-area metasurfaces. Recent work further extended NIL toward centimeter-scale functional meta-optical devices. As illustrated in Fig. 12c, meta-structures with lateral dimensions of approximately 5 mm × 5 mm were patterned while maintaining nanoscale linewidth control (~ 200–300 nm). Optical characterization confirmed uniform device response across the full aperture, indicating that large-area replication does not compromise optical functionality. These results highlight NIL’s ability to bridge nanoscale structural precision with macroscale optical component implementation [85].

**Fig. 13:**
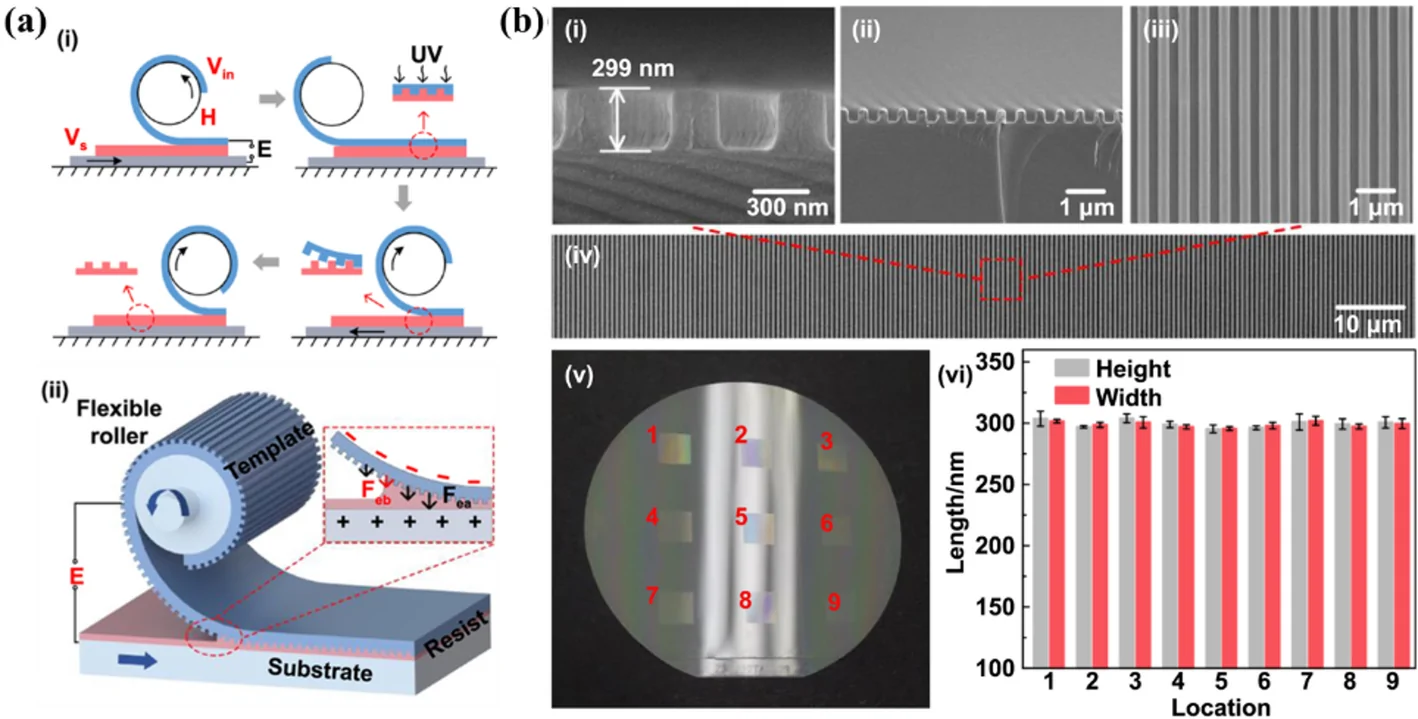
Flexible and R2R nanoimprint lithography for large-area photonics. **a** Schematic of UV-assisted R2R NIL process using a flexible template. **b** SEM images and large-area characterization of replicated sub-300 nm grating structures, showing high aspect-ratio profiles and uniform feature height/width distribution across multiple measurement locations. Adapted from Ref [87]. with permission

In parallel, R2R and flexible NIL processes have demonstrated continuous replication of sub-300 nm gratings over flexible polymer films. As shown in Fig. 13, UV-assisted R2R imprinting achieved line widths of ~ 300 nm and heights near 300 nm with uniformity variations below ± 5 nm across multiple sampled locations on the film [87]. Statistical analysis across nine measurement points showed height and width fluctuations within ~ 3–5%, confirming process stability over large areas. Cross-sectional SEM images reveal well-defined vertical sidewalls and minimal collapse, even for high-aspect-ratio features. Importantly, flexible NIL-defined nanostructures maintained structural integrity and optical performance under mechanical bending, supporting their integration into conformal photonic components. Compared with conventional stepper lithography, roll-based NIL can reduce cost per unit area while enabling meter-scale-equivalent pattern replication speeds, making it attractive for high-volume production of light-management films, flexible metasurfaces, and wearable optical devices. Taken together, these demonstrations confirm that NIL can deliver sub-300 nm resolution, wafer-scale uniformity, and large-area replication with dimensional variations typically within a few nanometers, while simultaneously supporting flexible substrates. Such quantitative performance metrics position NIL as a practical manufacturing platform for scalable and mechanically adaptable photonic systems. To achieve viable industrial deployment, however, continuous R2R NIL must overcome distinct engineering bottlenecks associated with web dynamics. Precise web-tension control is paramount to eliminate lateral structural distortion, while cumulative roller wear and mold contamination drastically limit stamp lifetime. Furthermore, the lack of real-time, non-destructive, large-area defect inspection methodologies remains a critical gap that hampers high-yield continuous manufacturing of flexible optical films.

### NIL in emerging CMOS device applications

**Fig. 14:**
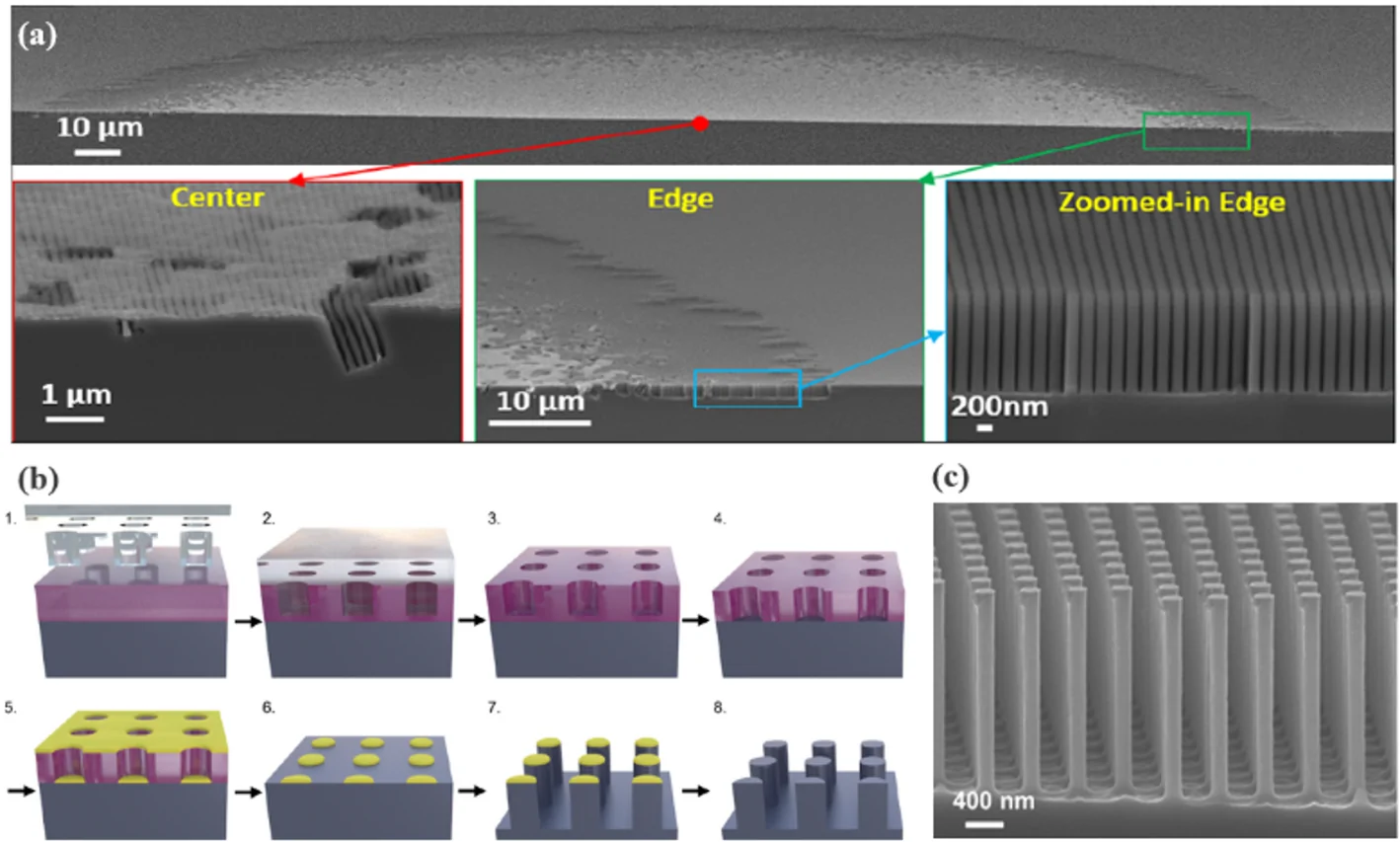
Nanoimprint-enabled nanofabrication: **a** optimized Ru-MacEtch for vertical Si fins. Adapted from Ref [88]. with permission. **b** Ge nanowire process flow using MgO masks. Adapted from Ref [89]. with permission. **c** ordered, size-controlled vertical Ge nanowire arrays. Adapted from Ref [89]. with permission

NIL has been explored as a high-throughput patterning route for selected 3D semiconductor nanostructures, although its broader use in advanced CMOS logic remains constrained by overlay, defectivity, and contamination requirements. In silicon applications, NIL is utilized alongside CMOS-compatible ruthenium-assisted chemical etching to overcome the limitations of plasma etching. By defining Ru mini-meshes through NIL and modulating catalytic activity with argon-carbon tetrafluoride plasma pretreatment, Mallavarapu et al. demonstrated the fabrication of nonporous, vertical silicon fins with aspect ratios exceeding 40, as shown in Fig. 14a [88]. Parallel developments in germanium technology leverage NIL and Bosch etching to realize highly ordered, catalyst-free vertical Ge nanowire arrays, with the fabrication sequence illustrated in Fig. 14b [89]. After the nanohole resist pattern was defined by NIL, MgO was deposited and lifted off to form a periodic hard-mask array. These MgO masks defined the Ge nanowires during subsequent Bosch etching and were removed after nanowire formation. This process allows precise control over nanowire height and enables diameter reduction to approximately 30 nm via wet etching, resulting in the high-density arrays shown in Fig. 14c [89]. Furthermore, NIL-defined Ge cores can be integrated into Ge-Si core-shell heterostructures. These structures facilitate hole gas accumulation within the high-quality Ge core, minimizing impurity scattering and providing a promising platform for next-generation vertical HEMT-type field-effect transistors.

## Advantages, limitations, and current challenges in NIL

The ascendance of NIL as a promising technology in the next-generation lithography landscape is underpinned by its unique physical mechanism, which offers several advantages over radiation-based patterning approaches. Unlike EUV or DUV lithography, NIL operates as a high-fidelity mechanical replication process, effectively decoupling the achievable resolution from the constraints of optical diffraction and depth-of-focus. This wavelength-independent nature allows the replication of sub-10 nm features using a comparatively simplified tool architecture. In addition, NIL exhibits a strong capability for single-step 3D nanopatterning, enabling the fabrication of complex multi-level structures such as blazed gratings, Fresnel lenses, and metasurfaces that would otherwise require multiple lithography and etching steps in conventional photolithography.

Despite these compelling strengths, the transition of NIL into mainstream semiconductor fabrication is tempered by several intrinsic technical and manufacturing limitations. As a contact-based technology, NIL is inherently susceptible to template-related issues, where the physical interaction between the mold and the resist can lead to defect propagation, template wear, and contamination. The requirement for 1:1 template-to-wafer replication means that any defect on the master mold is systematically transferred to every subsequent imprint, necessitating rigorous inspection and advanced cleaning protocols. Additionally, achieving nanometer-scale overlay alignment remains a critical challenge; mechanical distortions of the template and the complex fluid dynamics of the resist during the filling stage can induce local misalignments that complicate its integration into high-density CMOS logic flows. The management of the RLT also presents a bottleneck, as any non-uniformity across large-area wafers can lead to critical dimension variations during the subsequent RIE processes. Consequently, the practical impact of these limitations depends strongly on the target application. For photonic and optoelectronic devices such as metasurfaces, metalenses, micro-LEDs, and passive silicon photonic components, device performance is often governed primarily by nanoscale structural fidelity rather than extreme multilayer overlay accuracy. In contrast, advanced CMOS logic manufacturing imposes substantially more stringent requirements on defectivity, overlay precision, and contamination control, making large-scale NIL adoption more challenging despite recent advances in alignment and template technologies.

The application of NIL to specific semiconductor domains, such as power devices (SiC/GaN) and advanced optoelectronics, introduces a further layer of complexity related to substrate characteristics and integration workflows. In the context of wide-bandgap semiconductors, the inherent surface roughness and significant wafer bow or warp common in SiC and GaN substrates pose substantial hurdles to achieving uniform conformal imprinting. Incomplete cavity filling or localized pressure gradients can compromise the structural integrity of gate trenches or field plates in SiC MOSFETs. Moreover, the thermal expansion mismatch between the NIL template and these non-silicon substrates during thermal-NIL processes can induce parasitic mechanical stresses, potentially affecting device reliability. While NIL offers a promising pathway for the mass production of micro-LEDs and silicon photonic transceivers, its successful adoption depends on the development of specialized resist formulations and adaptive alignment systems capable of mitigating these substrate-specific anomalies while maintaining the economic scalability that defines the technology’s future potential.

## Future outlook and strategic opportunities

The future trajectory of NIL is increasingly defined by its transition from a specialized laboratory technique to an enabling platform for heterogeneous integration and non-conventional semiconductor manufacturing. As the industry moves toward the “More than Moore” era, NIL offers a scalable route for integrating electronic and photonic functionalities. One of the most significant opportunities resides in the rapid expansion of Artificial Intelligence (AI) infrastructure, which demands extreme data bandwidth and efficient thermal management. In this context, NIL may provide a practical manufacturing approach for high-density silicon photonics and CPO, where precise pattern replication is required for coupling gratings, optical interposers, and other nanophotonic structures.

Beyond high-performance computing, augmented reality (AR), virtual reality (VR), and mixed-reality optical systems represent additional opportunities for NIL-enabled manufacturing. The fabrication of metasurfaces and metalenses requires deterministic subwavelength patterning over relatively large areas, making manufacturing scalability a critical consideration. NIL has therefore emerged as one of the most promising fabrication approaches for these applications because it combines high-resolution pattern replication with the potential for large-volume production. Furthermore, increasing interest in sustainable manufacturing has motivated the exploration of NIL as a potentially lower-complexity patterning technology. However, the actual economic and environmental benefits depend strongly on process integration requirements, throughput, defectivity targets, and application-specific manufacturing conditions.

Looking ahead, the successful integration of NIL into the global semiconductor supply chain will depend on continued advances in template engineering, defect inspection, alignment technologies, and resist materials. Future manufacturing strategies may increasingly adopt hybrid lithography flows, in which NIL is selectively deployed for high-resolution three-dimensional structures and large-area nanophotonic devices, while conventional optical lithography remains responsible for the most overlay-sensitive device layers. Under such a framework, NIL is expected to complement rather than replace existing lithographic technologies, providing a valuable manufacturing pathway for next-generation photonic, optoelectronic, and More-than-Moore systems.

## Conclusion

Nanoimprint lithography has evolved from an alternative high-resolution lithographic concept into a versatile nanomanufacturing platform capable of combining nanoscale pattern precision with scalable fabrication. By decoupling pattern resolution from exposure wavelength, NIL enables deterministic replication of sub-100 nm and even sub-10 nm features while maintaining compatibility with wafer-scale or large-area processing. This fundamental advantage distinguishes NIL from conventional projection-based lithography and underpins its increasing relevance across multiple technological domains. The continued development of NIL process variants, including thermal NIL, UV-NIL, stepper-based imprint lithography, and R2R NIL has significantly expanded the technological capabilities of imprint-based fabrication. Each approach addresses distinct manufacturing requirements, ranging from high-fidelity nanostructure replication and semiconductor-compatible alignment to continuous large-area production. Concurrent advances in mold materials, resist chemistry, and surface engineering have further improved pattern fidelity, defect control, and process reliability. In recent years, NIL has demonstrated particularly strong potential in nanophotonic and optoelectronic applications. The ability to fabricate metasurfaces, metalenses, silicon photonic waveguides, microring resonators, and broadband antireflective structures with subwavelength precision highlights NIL’s capability to translate advanced optical concepts into manufacturable devices. Moreover, the scalability of imprint-based patterning offers a compelling pathway for high-volume production of photonic components required in emerging technologies such as optical interconnects, integrated photonic circuits, and large-area optical systems. Despite these advances, broader adoption still depends on resolving application-specific bottlenecks. For semiconductor-oriented fabrication, overlay accuracy, defectivity control, contamination management, and residual-layer-thickness uniformity remain critical, especially for multilayer or CMOS-related processes. For high-yield R2R NIL, template lifetime, mold wear, web-tension-induced distortion, and continuous defect inspection are central challenges. For silicon photonics, line-edge roughness, sidewall morphology, and etch-transfer fidelity must be controlled to reduce optical loss and device variability. Therefore, near-term opportunities are strongest in metasurfaces, metalenses, light-management layers, flexible photonics, passive silicon-photonic components, and selected More-than-Moore applications, where high-resolution replication and scalable manufacturing are more important than extreme multilayer overlay.

## Data Availability

No datasets were generated or analysed during the current study.
